# Design, Pharmacological Characterization, and Molecular Docking of Minimalist Peptidomimetic Antagonists of α_4_β_1_ Integrin

**DOI:** 10.3390/ijms24119588

**Published:** 2023-05-31

**Authors:** Monica Baiula, Michele Anselmi, Francesco Musiani, Alessia Ghidini, Jacopo Carbone, Alberto Caligiana, Andrea Maurizio, Santi Spampinato, Luca Gentilucci

**Affiliations:** 1 Department of Pharmacy and Biotechnology, University of Bologna, Via Irnerio 48, 40126 Bologna, Italy; monica.baiula@unibo.it (M.B.); andrea.maurizio2@unibo.it (A.M.); santi.spampinato@unibo.it (S.S.); 2 Department of Chemistry “G. Ciamician”, University of Bologna, Via Selmi 2, 40126 Bologna, Italy; michele.anselmi@iit.it (M.A.); alessia.ghidini@studio.unibo.it (A.G.); jacopo.carbone2@studio.unibo.it (J.C.); 3 Laboratory of Bioinorganic Chemistry, Department of Pharmacy and Biotechnology, University of Bologna, Viale Fanin 40, 40126 Bologna, Italy; francesco.musiani@unibo.it; 4 Department of Molecular Biology, Cell Biology and Biochemistry, Brown University, Providence, RI 02912, USA; alberto_caligiana@brown.edu; 5 Health Sciences & Technologies (HST) CIRI, University of Bologna, 40064 Ozzano Emilia, Italy

**Keywords:** VLA-4, leukocytes, peptidomimetics, antagonists, molecular modelling, inflammation

## Abstract

Integrin receptors mediate cell–cell interactions via the recognition of cell-adhesion glycoproteins, as well as via the interactions of cells with proteins of the extracellular matrix, and upon activation they transduce signals bi-directionally across the cell membrane. In the case of injury, infection, or inflammation, integrins of β_2_ and α_4_ families participate in the recruitment of leukocytes, a multi-step process initiated by the capturing of rolling leukocytes and terminated by their extravasation. In particular, α_4_β_1_ integrin is deeply involved in leukocyte firm adhesion preceding extravasation. Besides its well-known role in inflammatory diseases, α_4_β_1_ integrin is also involved in cancer, being expressed in various tumors and showing an important role in cancer formation and spreading. Hence, targeting this integrin represents an opportunity for the treatment of inflammatory disorders, some autoimmune diseases, and cancer. In this context, taking inspiration from the recognition motives of α_4_β_1_ integrin with its natural ligands FN and VCAM-1, we designed minimalist α/β hybrid peptide ligands, with our approach being associated with a retro strategy. These modifications are expected to improve the compounds’ stability and bioavailability. As it turned out, some of the ligands were found to be antagonists, being able to inhibit the adhesion of integrin-expressing cells to plates coated with the natural ligands without inducing any conformational switch and any activation of intracellular signaling pathways. An original model structure of the receptor was generated using protein–protein docking to evaluate the bioactive conformations of the antagonists via molecular docking. Since the experimental structure of α_4_β_1_ integrin is still unknown, the simulations might also shed light on the interactions between the receptor and its native protein ligands.

## 1. Introduction

The outer surface of the cell membrane presents receptors that mediate interactions between cells or with components of the extra cellular matrix (ECM). These interactions are fundamental in determining the scope and activities of the cell, providing physical, biochemical, and mechanical signals. These surface receptors are also connected to cytoplasmic proteins via their internal end, and can thus transmit messages bi-directionally between cells and their environment.

The most important family of membrane receptors that integrate the extracellular and intracellular environments via the transmission of signals between the two environments is represented by integrins, heterodimeric receptors composed of non-covalently linked α and β subunits. These receptors regulate fundamental functions, such as cell adhesion, signaling, and proliferation [1]. To date, 18 different α subunits and 8 different β subunits have been identified on cell membranes. Diverse combinations of an α and a β subunit give rise to 24 heterodimers; different heterodimers show not only a different cell expression profile but also different ligand binding properties and coupling to the cytoskeleton, and are therefore related to different signaling pathways [1,2].

Apart from the normal physiological functions, integrins are also deeply involved in a variety of diseases, including the initiation and progression of cancer, coronary diseases, and inflammatory and autoimmune pathologies. Consequently, antibodies or small molecules that modulate integrin–protein ligand interactions have attracted significant attention as potential drugs. Since 2015, more than 130 clinical trials of integrin-targeting medicines have been conducted [3,4].

Typically, the natural ligands of integrins are large proteins with relatively low binding affinities, i.e., glycoproteins expressed on the surface of other cells, such as the vascular cell adhesion molecule-1 (VCAM-1) and the intercellular adhesion molecule-1 (ICAM-1), or proteins of the ECM, including collagens, fibronectin (FN), vitronectin, laminins, and fibrinogen (Fg). Some integrins only bind to a particular ECM ligand, whereas other integrins are able to bind to several different ligands.

Remarkably, integrins recognize their endogenous ligands by interacting with very short peptidic sequences. The Arg-Gly-Asp (RGD) tripeptide is a highly conserved recognition motif found in ECM proteins, for e.g., in FN, vitronectin, and fibrinogen; is capable of binding to several integrins, including α_v_β_3_, α_v_β_1_, α_v_β_5_, α_v_β_6_, α_v_β_8_, α_5_β_1_, α_8_β_1_ expressed on cancer cells, and α_IIb_β_3_ integrin expressed on platelets.

Other integrins may bind to their ligands by recognizing different peptide motifs. In particular, the α_4_β_1_ integrin recognizes the Leu-Asp-Val-Pro (LDVP) peptide in FN, the Leu-Asp-Thr-Ser (LDTS) sequence in the mucosal addressing cell adhesion molecule-1 (MAdCAM-1), and Ile-Asp-Ser (IDS) in VCAM-1. This receptor, also known as very late antigen-4 (VLA-4), or as CD49d/CD29, plays a crucial role in the recruitment of leukocytes during inflammation, allergy, and autoimmune diseases, for e.g., multiple sclerosis (MS), but also in stem cell mobilization or retention in cancer development and metastasis [5].

α_4_β_1_ integrin is expressed in different types of cancer, including multiple myeloma, ovarian cancer, and pancreatic cancer, and recent studies have described its potential role in cancer development and metastasis formation. In thyroid cancer α_4_β_1_ is stabilized by DPP4 (dipeptidyl peptidase-4), a multifunctional cell surface glycoprotein with a negative prognostic value, leading to epithelial-to-mesenchymal transition and to tumor metastasis [6]. It has also been shown that activation of oncogenic-signaling pathways at least in part through α_4_β_1_ could exacerbate malignancy in pancreatic ductal adenocarcinoma [7]. In addition, a recent study has investigated the role of integrins in high-grade serous ovarian cancer, showing that α_4_ integrin can contribute to the metastatic spread of cancer cells and could also affect prognosis [8]. This receptor is also involved in the mechanism of resistance to bortezomib in multiple myeloma; accordingly, the rescue of high α_4_β_1_ expression and function confers resistance to proteasome inhibitors in multiple myeloma cancer cells [9,10].

Hence, targeting this integrin can be exploited for therapeutic purposes. The humanized monoclonal antibody (mAb), natalizumab, which targets α_4_β_1_ integrin, has already proven to be successful for the treatment of highly active relapsing and remitting MS [11]. Natalizumab was withdrawn from the market in 2005 because its use was associated with progressive multifocal leukoencephalopathy (PML) in patients with multiple sclerosis, but it was made available again the following year, albeit with a black box warning about the increased risk of PML. Moreover, it has been shown that allosteric activation of the leukocyte integrins α_4_β_1_ and α_L_β_2_ in T cells with the small molecule 7HP349 improved the penetration of cancer-specific T cells into tumors in mouse models of melanoma and colon carcinoma [12].

**Figure 1:**
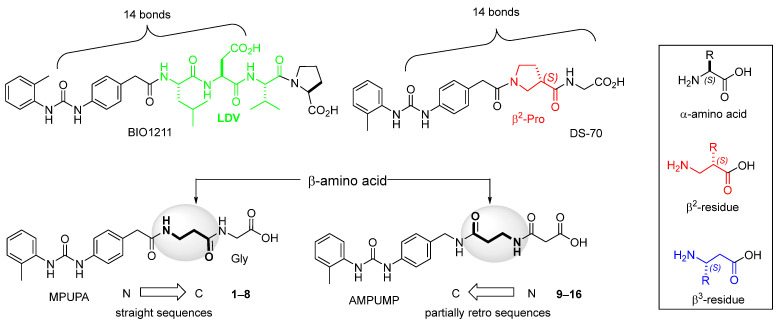
Structures of urea-peptide BIO1211, a potent antagonist of α_4_β_1_ integrin, of the peptidomimetic DS-70, containing a central β^2^-Pro core. Structures of hybrid α/β-peptides containing simple or functionalized β^3^- or β^2^-aminoacids: straight sequences **9**–**16** [25]; partially retro **9**–**16**.

As an alternative to antibodies, peptide ligands that reproduce the integrin-binding sequences can be utilized to interfere with integrin–ligand interactions [4]. The LDVP peptide BIO1211 (Figure 1) [13] was found to be a potent antagonist of α_4_β_1_ integrin capable of inhibiting antigen-induced airway hyper-responsiveness in allergic sheep [14]. This peptide owes its tremendous affinity to the α_4_-targeting diphenylurea pharmacophore. However, BIO1211 is scarcely stable in a biological environment, as tested in plasma; heparinized blood; and in homogenates of rat liver, lungs, and intestines [15,16], and is subjected to rapid clearance in vivo [17].

On the other hand, the stability of peptidic sequences can be improved by adopting a peptidomimetic strategy [18,19]. Several orthosteric peptidomimetic α_4_β_1_ integrin ligands have garnered much interest as antagonists for the treatment of asthma, [13] allergic conjunctivitis [20], or age-related macular degeneration (AMD) [21,22]. In addition, selective α_4_ integrin peptidomimetics were utilized to prepare monolayers of biofunctionalized nanoparticles and furnished cell-adhesive surfaces capable of detecting and quantifying leucocytes expressing active integrins, potentially useful for monitoring the severity and progression of correlated diseases [23,24,25].

From this perspective, in recent years we have explored the use of β-amino acids for improving the enzymatic stability of peptide integrin ligands [26]. Interestingly, while linear α/β-hybrid sequences were generally found to be antagonists [27,28,29], cyclic LDV pentapeptides including a β-residue proved themselves to be potent agonists, being capable of activating intracellular signalling and promoting integrin-mediated cell adhesion [30]. Among the linear structures containing a β-Pro scaffold, we found the potent α_4_ integrin antagonist DS-70 (Figure 1, Table 1) [20]. In contrast to BIO1211, the hybrid and minimalist α/β-peptidic structure conferred DS-70 noteworthy stability in mouse serum and significant in vivo efficacy [20,22].

Herein, we pursue the potential therapeutic use of the α/β-hybrid peptidomimetic α_4_β_1_ integrin ligands containing diverse simple or functionalized β^2^, or β^3^-amino acid cores (β-alanine, diaminopropionic acid [31], iso-aspartic acid), also in combination with the retro-sequence strategy [32]. The ability of the ligands to interfere with integrin functions was determined via cell adhesion assays, competitive binding assays, and by analyzing intracellular signaling.

Additionally, we discuss the plausible ligand–receptor interactions using molecular modelling studies. Since the three-dimensional structure of α_4_β_1_ integrin is not yet available, the study of a ligand’s structural determinants for agonism/antagonism at this integrin is of great interest to the development of a reliable pharmacophoric model for future drug design.

## 2. Results

### 2.1. Peptidomimetics Design and Synthesis

Starting from the peptides DS-70 and DS-23, which contain a central β^2^-Pro core [20], we derived a mini-library of previously described straight sequences **1**–**8** [25], as well as partially retro sequences **9**–**16**. The straight sequences **1**–**8** of general structure MPUPA-β-residue-glycine are *N*-capped with the o-methylphenylureaphenylacetic acid (MPUPA) moiety, while the partially retro sequences **9**–**16** of general structure AMPUMP-β-residue-malonic acid include the 1-(4-(aminomethyl)phenyl)-3-(o-methylphenyl)urea group (AMPUMP).

β-amino acids carrying the side chain adjacent to the carboxylic acid group are termed β^2^, or β^3^ when the side chain is located adjacent to the β-amino group (Figure 1) [33,34]. The β-residue consents to maintain the same span of 14 bonds from the urea carbonyl to the carboxylate of Gly as the distance between the urea carbonyl and the carboxylate of Asp in BIO1211 (Figure 1). The presence of a carboxylate group is generally regarded as a mandatory requisite for a large majority of integrin ligands [1,5,23] (see also Section 2.6).

The peptide mimetics were prepared in solution or in solid phase under standard conditions, as reported in Materials and Methods and Supporting Information. The sequences **1**, **2**, **5**, **6**, and **9**–**16** were prepared in solution, while the remaining sequences were prepared with solid-phase extraction. Although the syntheses of sequences **1**–**8** have been already described [25], the fundamental synthetic steps are outlined for thoroughness. The MPUPA moiety was prepared (Supplementary Materials) via condensation of o-tolylisocyanate and 2-(4-aminophenyl)acetic acid. AMPUMP was prepared via a similar protocol and from Boc-4-(aminomethyl)aniline (Supplementary Materials, Scheme S1). As for the β-residues, we prepared simple or functionalized β^2^ or β^3^-amino acids in-house (see Materials and Methods and Supporting Information).

In general, after synthesis the crude peptides were purified using semipreparative RP HPLC over a C18 column (General Methods) using H_2_O/acetonitrile mixtures containing 0.1% trifluoroacetic acid; purity was confirmed to be >95% via RP HPLC; structure identity was assessed via ESI-MS and NMR spectroscopy.

### 2.2. Effects of Peptidomimetics on Integrin-Mediated Cell Adhesion

To investigate the ability of the new peptides to modulate integrin-mediated cell adhesion, we employed cell adhesion assays using a Jurkat E6.1 cell line, which endogenously expresses α_4_β_1_ integrin and the natural ligand FN. Cell adhesion experiments allowed us to identify compounds defined as antagonists, i.e., those capable of reducing the number of adherent cells bound to an integrin endogenous ligand. On the contrary, ligands able to increase cell adhesion are defined as agonists.

DS-70 and BIO1211 were considered as reference antagonists [20]. Regarding the new peptides, the potencies of the retro peptides **9**–**16** (Table 2) were found to be comparatively inferior to those of the ligands with straight sequences **1**–**8** (Table 1) [25]. Concentration–response curves are shown in Supporting Information (Figure S1). Straight sequence **8** was shown to be an antagonist of α_4_β_1_ integrin with an excellent potency, comparable to those of the reference compounds DS-70 and BIO1211 (Table 1). In addition, peptidomimetic **3** and retro sequence **13** were able to reduce α_4_β_1_-mediated cell adhesion, behaving as antagonists, with IC_50_ values in the submicromolar range (Table 1 and Table 2).

The peptides that showed the most relevant activity (Table 1 and Table 2) were selected for determining their selectivity to different integrins (Table 3; concentration–response curves are shown in Supporting Information, Figures S2–S4). To this purpose, the ability of the best peptidomimetics to modulate cell adhesion mediated by integrins other than α_4_β_1_ was evaluated in HL60 cells, mainly expressing the leukocyte integrin α_M_β_2_, and on K562 cells, mainly expressing α_5_β_1_, which shares a β_1_ subunit with the heterodimer α_4_β_1_. Interestingly, peptide **3** strongly reduced the adhesion of HL60 cells to the natural ligand Fg, showing excellent potency. In contrast, **13** was a moderate promoter of cell adhesion mediated by α_M_β_2_ integrin, behaving as an agonist. Regarding α_5_β_1_ integrin, none of the peptidomimetics employed were able to modulate K562 cell adhesion, thus being shown to be completely ineffective toward α_5_β_1_ integrin. The reference compound DS-70 was ineffective toward both α_M_β_2_ and α_5_β_1_ integrins under the same experimental conditions (Table 3).

### 2.3. Evaluation of Binding Affinity to α_4_β_1_ Integrin

To better characterize α_4_β_1_ integrin–peptidomimetic interaction, competitive binding experiments on intact Jurkat E6.1 cells were performed; thus, measuring the ability of increasing concentrations of the new compounds to displace the labelled ligand LDV-FITC from α_4_β_1_ integrin (Table 4). The measured binding affinity of the reference compounds BIO1211 and DS-70 was comparable to the previously published IC_50_ for ligand binding [30], being K_i_ 6.9 ± 3.1 nM and 0.94 ± 0.32 nM, respectively.

Regarding the straight hybrid peptides, only compounds **3** (K_i_ = 1.3 ± 0.7 nM) and **8** (K_i_ = 1.9 ± 0.8 nM) were able to bind to α_4_β_1_ integrin, showing excellent affinity. The competitive binding experiments performed on partially retro hybrid peptides showed that only peptide **13** binds with very good potency to the receptor (K_i_ = 16.7 ± 5.2 nM).

### 2.4. Effects of Peptidomimetics on α_4_β_1_ Integrin Conformation

Integrin functions are regulated by a number of conformational changes in the protein itself. Three major conformations have been described: an inactive or bent conformation, an intermediate-activity conformation, and an open high-activity conformation [35]. Conformational changes can be evaluated by using conformationally sensitive antibodies which specifically recognize epitopes exposed only in a defined structural conformation. To better depict peptidomimetics–α_4_β_1_ integrin interactions, we employed PE-conjugated HUTS-21 mAb that recognizes an epitope mapped to the hybrid domain of the β_1_ subunit [36]. This epitope is a LIBS (ligand-induced binding site epitope) which is hidden in the inactive bent conformation but is exposed in the intermediate-activity and open high-activity conformation, upon agonist binding or partial integrin activation.

**Figure 2:**
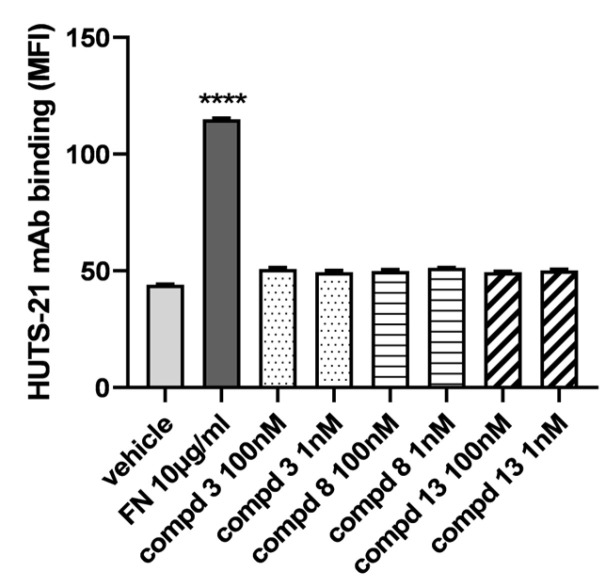
Evaluation of HUTS-21 antibody binding to Jurkat E6.1 cells in the presence of different concentrations of the new peptidomimetics. Jurkat E6.1 cells were incubated with FN (10 µg/mL) or the most active compounds (**3**, **8**, and **13**; 1–100 nM), and HUTS-21 mAb was then added to measure the effects of the selected ligands on exposure of the HUTS-21 epitope to α_4_β_1_ integrin. Antagonists **3**, **8**, and **13** were not able to increase the binding of HUTS-21 mAb. Results are expressed as mean fluorescence intensity (MFI) ± SD of three independent experiments carried out in duplicate. MFI values for respective isotype control mAb were set to 0. **** *p* < 0.0001 versus vehicle (Tukey’s test after ANOVA).

To evaluate the effects of the most active peptidomimetics (**3**, **8**, and **13**), Jurkat E6.1 cells were pre-incubated with different concentrations (1–100 nM) of the ligands, then PE-HUTS-21 mAb was added and fluorescence was measured using flow cytometry. As expected, the α_4_β_1_ endogenous ligand FN induced a conformational change in α_4_β_1_ integrin, leading to the exposure of HUTS-21 epitope and thus to the increased binding of the HUTS-21 mAb, with respect to vehicle-treated cells that have α_4_β_1_ integrin mainly in the inactive conformation (Figure 2). Conversely, antagonists **3**, **8**, and **13**, although binding to α_4_β_1_ integrin as previously shown, were not able to induce any conformational rearrangement, showing very low binding of HUTS-21 mAb.

### 2.5. Effects of Peptidomimetics on Integrin-Mediated Intracellular Signaling

Integrins relay signals across cell membrane, functioning as integrators of the ECM or other cells-driven cues. Since integrin cytoplasmic tails are short and lack kinase activity, integrin transduces signals within the cell, facilitating the assembly of cytoplasmic adaptor and/or signaling proteins in large complexes named focal adhesions (FAs). FAs are intracellular signaling platforms that enable cells to respond to changing extracellular signals by modulating downstream long-term events, including cell proliferation, differentiation, and migration [37]. Among the different signaling pathways activated by integrins, the MAPK and AKT pathways play an important role.

**Figure 3:**
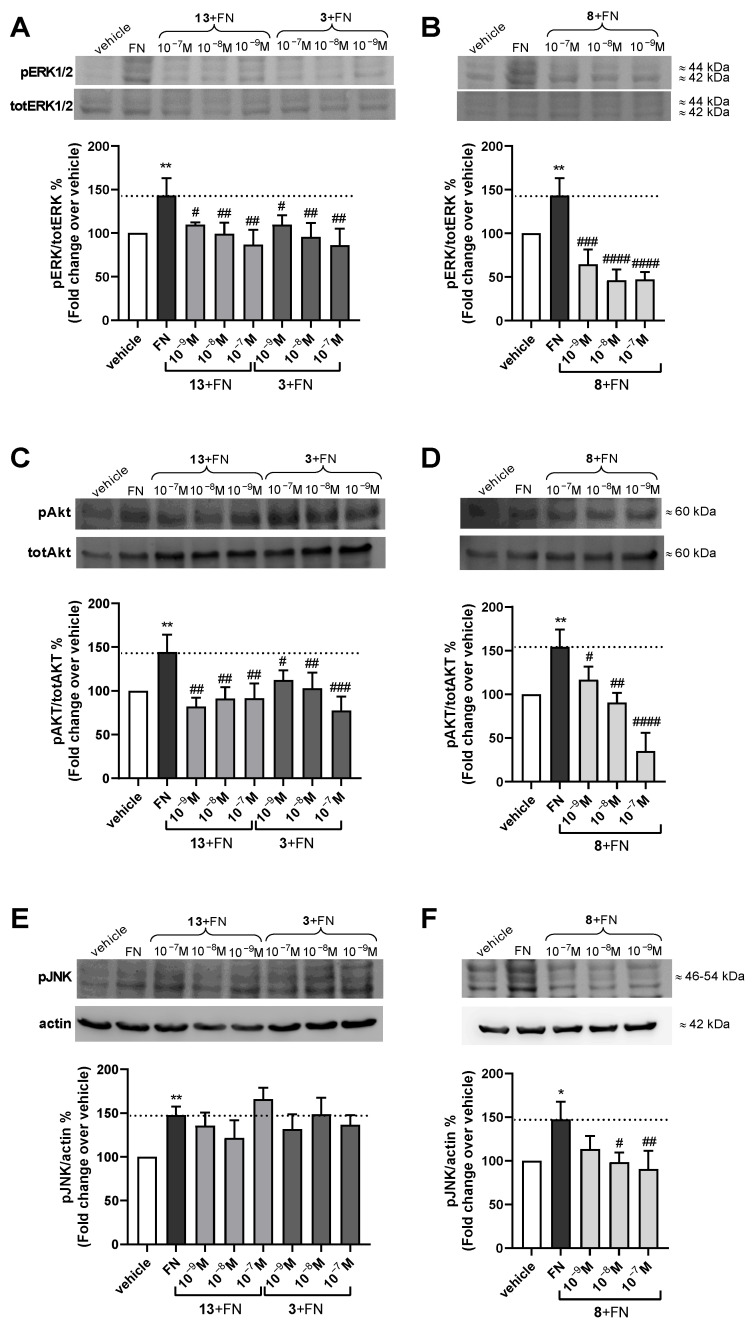
Effects of the most effective peptidomimetics on integrin-mediated intracellular signaling. Integrin antagonists **3**, **13**, and **8** were able to significantly prevent FN-induced α_4_β_1_ integrin-dependent activation of ERK1/2 (**A**,**B**) and AKT (**C**,**D**). Conversely, **3** and **13** did not block JNK activation induced with FN (10 µg/mL) (**E**), whereas **8** significantly reduced FN-induced JNK phosphorylation (**F**). The representative Western blot shows that Jurkat E6.1 cells plated on FN had a stronger signal for pERK1/2, pAKT, and pJNK than vehicle-treated cells (vehicle). The graphs represent densitometric analysis of the bands (mean ± SD; three independent experiments). The amount of phosphorylated kinases (pERK1/2 or pAKT) was normalized to that of the corresponding total kinase (totERK1/2 or totAKT); phospho-JNK (pJNK) was normalized to actin. * *p* < 0.05, ** *p* < 0.01 vs. vehicle; # *p* < 0.05, ## *p* < 0.01, ### *p* < 0.001, #### *p* < 0.0001 vs. FN (Tukey’s test after ANOVA).

To further characterize the most promising peptidomimetics, **3**, **8**, and **13**, their effects on integrin-mediated AKT and MAPK signaling pathways were evaluated using a Western blot (Figure 3).

To this purpose, Jurkat E6.1 cells were pre-treated with different concentrations (10^−7^, 10^−8^, 10^−9^ M) of the compounds and then exposed to FN (10 µg/mL) for 30 min. As shown in Figure 3, the FN was able to induce integrin-mediated intracellular signaling activation through the phosphorylation of ERK1/2, AKT, and JNK. When Jurkat E6.1 cells were pre-exposed to **3**, **8**, or **13**, all three α_4_β_1_ ligands significantly prevented FN-induced ERK1/2 and AKT phosphorylation. In addition, the effects observed on intracellular signaling were concentration-dependent, except for compound **13** on AKT activation.

Conversely, regarding JNK activation, only peptidomimetic **8** was able to significantly prevent FN-induced JNK phosphorylation, whereas **3** and **13** did not counteract FN-activating effects on JNK intracellular signaling pathways.

Overall, these data confirm that peptidomimetics **3**, **8**, and **13** behave as α_4_β_1_ integrin antagonists, being able to bind to the receptor with excellent affinity but without inducing any conformational switch. Therefore, through their direct interaction with the receptor, they are able to inhibit cell adhesion to integrin endogenous ligands and to prevent FN-induced activation of intracellular signaling pathways, thus blocking α_4_β_1_ integrin functions that are involved in the pathogenesis of several diseases, including different types of cancer and inflammatory diseases. Nevertheless, further studies are needed to better unravel the detailed mechanisms by which peptidomimetic antagonists block α_4_β_1_ integrin functions.

### 2.6. Molecular Docking

The 3D structure of α_4_β_1_ integrin has not been experimentally disclosed yet. However, both the α_4_ and β_1_ subunits have been determined in several separate heterodimeric complexes throughout X-ray crystallography and cryo-EM. Consequently, an α_4_β_1_ integrin structure model can be obtained by employing molecular modelling techniques. In this work, the model structure of human α_4_β_1_ integrin was obtained through protein–protein docking, using α_4_ and β_1_ monomers taken from experimental dimeric structures of integrins α_4_β_7_ (PDB ID 3V4V) and α_5_β_1_ (PDB ID 4WK0), respectively. The docking calculation was guided using the conserved residues found at the interfaces in the experimental integrin dimeric structures (Figure S5). The resulting model was in good agreement with other integrin structures and shows a large interaction surface between the two subunits (1733 Å^2^). The metal ions binding sites were optimized using a known modelling procedure [38,39] in order to be as close as possible to equivalent sites present in the experimental structures.

**Figure 4:**
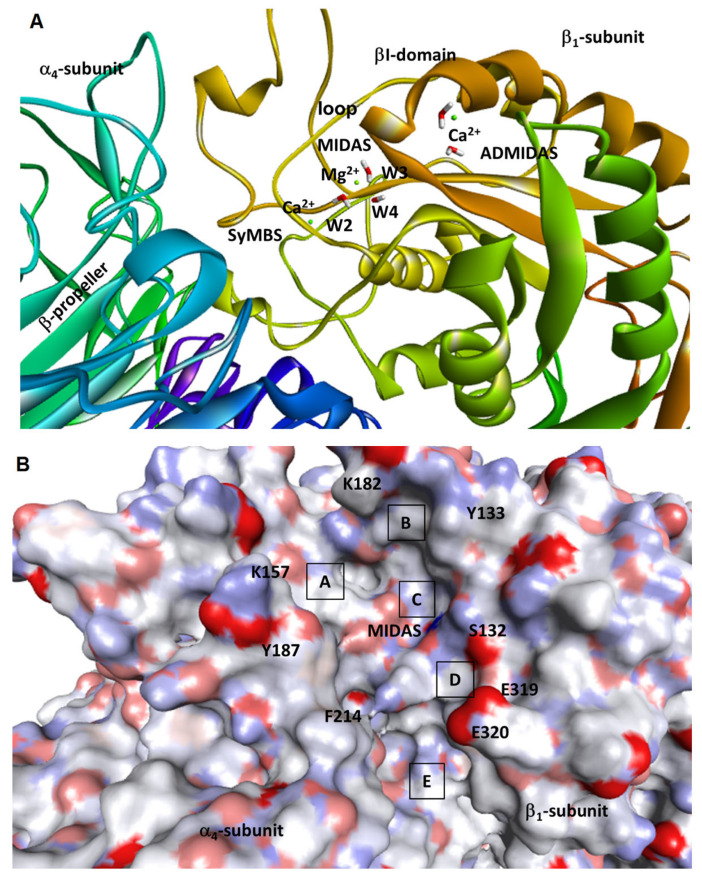
(**A**) Ribbon image of the binding site of the model of α_4_β_1_ integrin, showing Ca^2+^ cations in SyMBS and ADMIDAS, and Mg^2+^ in MIDAS. The water molecules W2–4 are also shown, while W1 was depleted to make space for the ligands (Supporting Information). (**B**) Solvent accessible surface, showing subpockets A–E; the positions of relevant residues are also shown.

The α_4_ subunit appears remarkably different from the other α subunits of RGD-binding integrins, since it completely lacks in the cavity deputed to host the ligand’s arginine. In our homology model, the supposed binding site is delimited by the βI domain of the β subunit and the β-propeller in the α subunit (Figure 4A). In the β_1_ subunit, the βI domain contains a Mg^2+^ ion in the metal ion dependent adhesion site (MIDAS), fundamental to the interaction with negatively charged ligands (Figures S6 and S7, and Table S1).

Most of the known integrin ligands share a common carboxylate group as the fundamental pharmacophore. In addition, two other metal centers are present (Figure 4A and Figure S6) that contain one Ca^2+^ metal ion each, the “adjacent to metal ion-dependent adhesion site” (ADMIDAS) and the “synergistic metal ion-binding site” (SyMBS) (for ADMIDAS: Figure S8 and Table S2; for SyMBS, Figure S9 and Table S3). In α_5_β_1_ integrin, these metal centers play a regulatory role in ligand binding. Additionally, the inspection of the solvent-accessible surface of the model revealed that the putative binding site is formed by subpockets A–E, characterized by diverse dimensions, shapes, and delimiting residues (Figure 4B).

In the so-obtained model of the α_4_β_1_ integrin, the coordination sphere of Mg^2+^ ion at the MIDAS includes four water molecules (W) and the residues Ser132(β) and Glu229(β). As reported [1], an orthosteric, negatively charged ligand is expected to form a salt bridge with Mg^2+^ ion at the MIDAS by substituting one of the four water molecules (Figure S10). In the X-ray crystallographic structures of integrin α_5_β_1_, which are used as templates for the β subunit (PDB ID: 4WK0), as well as in the analogous structures (PDB IDs: 4WK2 and 4WK4), the ligands always occupy the position labelled as W1 (Figure S10).

The first series of docking experiments was carried out to understand whether that position is the only one of the first metal coordination spheres accessible to the ligand. Different docking calculations were performed with the same parameters, each time eliminating a different water molecule from the first coordination sphere of Mg^2+^ to make it accessible to the ligand. In particular, the MIDAS water molecules at positions W1, W2, and W3 were alternatively removed, while the water molecule at position W4 was maintained because it is not accessible to the bulk of the solvent (Supporting Information). The simulation was repeated using different ligands, a process already reported in the literature. In general, the removal of W1 allowed the ligands to coordinate Mg^2+^ ion, while in the other cases the ligands could not get close enough to the metal center. Consequently, the docking calculations were conducted by eliminating water and leaving position W1 accessible to potential ligands (Figure 4A).

For the computations, we selected the hybrid peptides exhibiting at least a measurable efficacy, i.e., DS-23, DS-70, **8**, **3**, **13**, and the reference antagonist BIO1211, as well as structurally correlated compounds with modest-to-null activity (**7**, **5**, **1**, **2**, **4**, **14**, **10**), for comparison. The aim of the computations was to highlight the specific structural determinants of ligand binding.

Each ligand was minimized and parametrized (see Section 4, and Figure S13); special attention was paid to modelling and optimizing the structures of the diphenylurea and β-Pro fragments (Figures S11 and S12). Then, the ligands were docked into the α_4_β_1_ integrin model achieved in the previous modelling step.

The best docking poses of each compound exhibiting good potency during in vitro studies were used to define the main interactions between these antagonist ligands and α_4_β_1_ integrin, and thus the main amino acid residues involved. The complexes between each one of these poses and α_4_β_1_ were analyzed with the web server PacDOCK [40] and the program LigPlot+ [41]. The main integrin residues involved in the binding of these compounds are reported in Figure S14. In all the best docked poses, every compound interacts with both residues from the β and α subunit. The amino acid residues more often involved in the interaction are Ser132, Gly223, Asn224, Leu225, Asp226, Ser227, and Glu229 of the β subunit, and Tyr187 and Phe214 of the α subunit.

**Figure 5:**
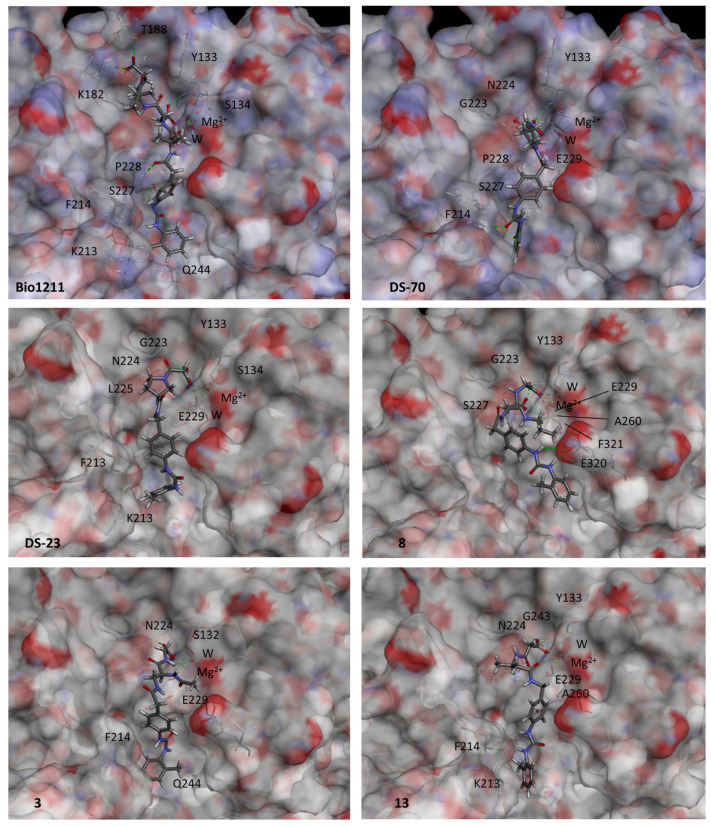
Calculated binding conformations of hybrid ligands within the α_4_β_1_ integrin binding site. Ligands are rendered in stick and colored by atoms (C, grey; N, cyan; O, red; H, white). The integrin binding site is represented by its partially transparent, solid solvent-accessible surface, colored by the atomic interpolated charge. Key receptor residues are represented in tiny sticks, and nonbonding interactions are indicated as dashed lines. Images were obtained using BIOVIA DSV2021.

To improve the accuracy in the definition of the docked poses and to identify the best pose for each good ligand, a re-scoring of all the most representative docked poses was performed using the AMBER score implemented in the program UCSF DOCK 6 and based on the MM-GBSA method (see Section 4 for more details on the procedure). The best docked poses according to the AMBER score for all the compounds with significant potency, BIO1211, DS-70, DS-23, **8**, **3**, and **13,** are reported in Figure 5.

In the best pose predicted for BIO211, MPUPA-Leu-Asp-Val-ProOH, the peptidic sequence lies vertically along the α4β1 crevice (Figure 5), using subpockets B–E as defined in Figure 4B. Ligand’s Asp is well inserted into subpocket C, so that the main interaction is the salt bridge between Asp carboxylate and Mg^2+^. AspCOO^−^ also interacts with other residues of the MIDAS region, Ser132 and Tyr133.

As for the rest of the ligand’s structure, the peptidic portion attains interactions only with the β_1_ subunit; in particular, the side chains of Leu and Val occupy subpockets D and B, respectively. In subpocket B, Pro carboxylate makes a salt bridge with Lys182(β1) and also interacts with Thr188(β1). The diphenylurea is longitudinally inserted within subpocket D, showing stabilizing contacts with residues of the α subunit, i.e., Gln244, Lys213, and Phe214, as well as residues from the β subunit, Ser227, Pro228.

Very recently, the docking of BIO1211 into a homology model of the α_4_β_1_ integrin was simulated by da Silva et al. [42]. The proposed bioactive conformation has something in common with the structure shown in Figure 5, the main difference being the orientation of the C-terminal Val-Pro dipeptide, which in da Silva’s geometry is rotated towards the A subpocket, so that Pro cannot reach Lys182(β_1_).

Concerning the much shorter sequences of the hybrid peptides, for the compounds with a significant activity the docked poses reproduced the position of the diphenylurea-Leu-Asp portion of BIO1211, occupying subpockets C, D, and E. In particular, for each compound the negatively charged carboxylate entered into C and formed a salt bridge with Mg^2+^ at the MIDAS, while the diphenylurea moiety found a place in lower subpocket E of the α/β crevice (Figure 5).

Interestingly, simulations of compounds exhibiting scarce potency in vitro failed to reproduce the fundamental interaction between the ligands’ carboxylate and the MIDAS. For some of these compounds, i.e., **2**, **5**, **9**, and **14**, poses have been identified in which the diphenylurea fits subpocket E and the carboxylate is oriented towards the MIDAS; however, in general the distance between the carboxylate and Mg^2+^ is greater than 3 Å (Supporting information, Figure S15).

Apparently, the cyclic β^2^-Pro scaffolds allow DS-70 and DS-23 to optimally direct the carboxylate and diphenylurea pharmacophores across the binding site, allowing the backbones to pass over the saddle that separates subpockets C and E (Figure 5).

The simulation of potent antagonist **8** revealed that the (*R*)-isoAsp β^3^-core nicely fits subpocket D (see Figure 4B) and is delimited by residues of the β_1_ subunit, i.e., Phe321, Ala260, and Ser227. The profound insertion of the propylamide side chain into pocket D seems to pull the entire structure against the β_1_-subunit, so that both urea NHs of MPUPA in subunit E make a bifurcated hydrogen bond with E320, while Gly carboxylate is tightly bound to MIDAS (salt bridge) and to the β_1_ residues Tyr133, Gly223, and Asn224.

The computations suggest that the comparatively inferior affinity of **3** might depend on the modest contribution to complex stabilization from *N*-acetyl (*S*)-Dap (diaminopropionic acid) scaffold access into subpocket D. GlyCOO^−^ can still bind to the MIDAS and interact with other elements of subpocket C, Asn224, and Ser132, but the diphenylurea seems to lose most of the above-mentioned stabilizing contacts within the E pocket. This interpretation seems in line with the complete lack of receptor affinity of **1**, topologically correlated to **3**, since the former addresses subpocket D with a simple methyl (Figure S15).

Regarding the calculated pose of **13**, although the pharmacophores are still able to attain the necessary positions within the binding site, the (*R*)-β^3^-Ala scaffold completely lacks any interactions with the D subpocket, plausibly explaining the very low efficacy of this ligand.

## 3. Discussion

Integrins are involved in many biological processes, such as cell adhesion and migration, and are responsible for the survival of the cell and the control of the cell cycle. In addition, integrins play a fundamental role in several diseases, including cancer progression and metastasis, coronary disease, thrombosis, fibrosis, inflammatory, and autoimmune pathologies. It is precisely their involvement in the main human biological process and pathobiological processes of various diseases that render integrins very attractive therapeutic targets. To date, six different integrin-targeting drugs have been marketed, and many others are under investigation [3,4,5].

A recent research topic concerns the development of inhibitor peptides, associated with many advantages, such as higher specificity and efficacy with respect to small molecules, and lower immunogenicity and costs with respect to macromolecules [3,5,13,22,23]. Nevertheless, native peptides are associated with many limitations, such as low metabolic stability toward proteolysis and poor absorption. To improve these limited properties, natural peptides can be structurally modified, obtaining compounds called peptidomimetics. Most of the peptides entering clinical trials today are, in fact, peptidomimetics.

To increase the bioavailability of integrin ligands, we opted for small hybrid α/β-peptidomimetics. In particular, the compound DS-70, MPUPA-β^2^Pro-Gly, and its partially retro analogue DS-23, were found to be potent antagonists of this receptor [20]. Starting from these ligands, derivatives **1**–**16** were designed by introducing diverse β^2^- or β^3^-residues, in either an (*S*)- or (*R*)-configuration, and straight sequences **1**–**8** [25] and partially retro-sequences **9**–**16** were tested in vitro in integrin-mediated cell adhesion experiments to understand their effects.

As it turned out, the ligands DS-70, DS-23, **3**, **8**, and **13** were able to bind with excellent affinity to the receptor. All these compounds strongly reduced the adhesion of integrin-expressing cells to the natural ligands, without inducing receptor open conformation nor activation of intracellular signaling pathways. Intriguingly, DS-70, DS-23, and **8** showed very high potency, with IC_50_ values in a low nanomolar range, similar to that of the reference peptide BIO1211, despite the much smaller structure.

This observation provoked interest in the potential mechanisms that form the basis of the efficacy toward α_4_β_1_ integrin. To investigate the chemical and structural determinants that lead to integrin binding and inhibition, some representative compounds were analyzed via molecular docking. The α_4_β_1_ integrin dimer was obtained from the structures of the separate monomers, and the resulting binding site appeared to be partitioned in subpockets A–E, which were of different sizes and compositions (Figure 4B). For the reference BIO1211, the simulations revealed a perfect fit into subpockets B–E of the α/β crevice (Figure 4B and Figure 5), giving rise to many interactions, including salt bridges involving Asp and Pro carboxylates. As for the minimalist hybrid ligands, the calculations were suggestive of bioactive poses that in part reproduce the binding of BIO1211 by occupying subsites C, D, and E. In particular, the latter nicely hosts the diphenylurea group, confirming the efficacy of this α_4_ integrin-targeting motif [13].

Interestingly enough, the simulations highlighted the fundamental role of the β-residues, their configuration, and the specific side chains. Indeed, for DS-70 and DS-23, high affinity for the receptor seems to be correlated to the ability of the cyclic β^2^-Pro scaffold to pass over the saddle between pockets C and E and to orient the pharmacophores toward the respective subpockets.

For the bioactive compounds which include the linear β^2^- or β^2^-residues, the results in general were inferior in respect to DS-70 and DS-23, with the exception of **8**, characterized by a low nanomolar affinity. The comparison of the binding poses of **8**, **3**, and **13** support that good receptor affinity seems to correlate to the ability of the central β-residue to place its side chains into subpocket D. This would consent the ligands to maintain at least in part the network of interactions of the reference LDVP peptide BIO1211, including the fundamental interaction, i.e., the ionic bond between negatively charged carboxylate with the MIDAS Mg^2+^ ion of the β subunit, with a distance comparable to those found in X-ray crystallographic structures (between 1.92 and 2.04 Å).

In contrast, this direct coordination was not maintained for compounds associated with an inferior in vitro potency. Although docked poses in which the carboxylate is oriented toward the Mg^2+^ of the MIDAS have been identified for some of the low potency compounds, the distance was >3 Å (Figure S15).

Although the computational and experimental evidence seems in good agreement, it must be stressed that, as with any theoretical study, our model has some limitations. The accuracy that can be expected from homology modeling is highly dependent on the sequence identity between the target and templates. In the present case, the model structure of α4β1 integrin was derived from the experimental structures of the α_4_ and β_1_ monomers bound to different partner subunits. We assumed that the tertiary structure of each monomer was the best available, while the relative orientation of the two monomers and the conformations adopted by the side-chains of the residues at the interface may be less accurate. Fortunately, the largest part of the binding pocket was maintained unaltered during the protein–protein docking procedure. The metal binding sites were the subject of an accurate refinement in order to reproduce the experimental coordination geometries.

For these reasons, we felt reasonably confident to perform protein–ligand docking. For the subsequent docking calculations, we relied on scoring functions to rank and select the best binding poses. Diverse docking/scoring methods are available to provide reasonable predictions of ligand binding modes, but their performances are often system-dependent [43]. Hence, we conducted preliminary redocking calculations to establish the best docking procedure to be used in the case of integrins.

To conclude, on the basis of the experimental and theoretical evidence, we believe that our findings support that α_4_β_1_ integrin antagonists may represent useful research tools to develop more effective drugs to fight cancer and inflammatory diseases. Nevertheless, further studies are needed to better unravel the mechanisms by which integrin peptidomimetic antagonists block integrin functions, including the analysis of adhesion kinetics, the effects under repulsive forces, and the formation of focal adhesion. Put in perspective, more functional assays need to be employed to better investigate the anti-inflammatory and/or anti-cancer effects of α_4_β_1_ integrin peptidomimetic antagonists.

## 4. Materials and Methods

### 4.1. General Methods

Chemicals and solvents were purchased from commercial sources and used without further purification. Peptides were purified via semipreparative RP HPLC under the following conditions: Agilent 1100 series apparatus; reverse-phase column Waters XSelect Peptide CSH C18 OBD column, 19 × 150 mm 5 μm (column description: stationary phase octadecyl carbon chain-bonded silica, double-end-capped, particle size of 5 μm, pore size of 130 Å, length of 150 mm, internal diameter of 19 mm; DAD of 210 nm, DAD of 254 nm); mobile phase isocratic 1:1 H_2_O/CH_3_CN/0.1% TFA, flow rate 10 mL/min^−1^.

The purities of peptide ligands were determined to be ≥95% via analytical HPLC analyses under the following conditions: Agilent 1100 series apparatus; reverse-phase column Phenomenex mod. Gemini 3 μm C_18_ 110 Å 100 × 3.0 mm (stationary phase octadecyl carbon chain-bonded silica—trimethylsilyl endcap, fully porous organosilica solid support, particle size of 3 μm, pore size of 110 Å, length of 100 mm, internal diameter of 3 mm); mobile phase: from 9:1 H_2_O/CH_3_CN/0.1% HCO_2_H to 2:8 H_2_O/CH_3_CN/0.1% HCO_2_H in 20 min, flow rate of 1.0 mL/min^−1^; DAD of 254 nm.

ESI MS analysis was carried out under the following conditions: MS single quadrupole HP 1100 MSD detector, drying gas flow of 12.5 L/min^−1^, nebulizer pressure of 30 psig, drying gas temp. of 350 °C, capillary voltages of 4500 (+) and 4000 (−), scan range of 50–2600 amu. NMR spectra were recorded using Varian Gemini apparatus, for ^1^H NMR, at 400 MHz, and for ^13^C NMR, at 100 MHz, at 298 K in 5-mm tubes, using 0.01 M peptide. Solvent suppression was carried out via the solvent presaturation procedure implemented in Varian (PRESAT). Chemical shifts are reported in ppm (δ) with the following internal standards: CDCl_3_ ^1^H NMR 7.26 ppm, ^13^C NMR 77.16 ppm; (CD_3_)_2_SO ^1^H NMR 2.50, ^13^C NMR 39.52 ppm. The assignment of ^1^H NMR resonances was based on 2D gCOSY experiments.

### 4.2. Synthetic Procedures

The synthesis and isolation of compounds **1**–**8** has been reported elsewhere [25]. For convenience, the synthetic steps are resumed in Supporting Information, together with the synthetic schemes for **9**–**16**.

The MPUPA moiety was prepared via reaction between o-tolylisocyanate and 2-(4-aminophenyl)acetic acid, while AMPUMP was prepared from tert-butyl (4-aminobenzyl)carbamate and o-tolyl isocyanate, followed by Boc cleavage (Supplementary Materials, Scheme S1).

(*S*)- or (*R*)-Boc-β3-homoAla-OH was prepared via the homologation of (*S*)- or (*R*)-Boc-Ala-OH, as reported by Caputo et al. (Scheme S2) [44]. (*S*)- or (*R*)-β2-Ala was obtained as methyl ester by using a modified version of a multi-step procedure as reported by Lee et al. (Scheme S3) [45]. (*S*)- or (*R*)-Fmoc-isoAsp(NHPr)-OH was prepared via coupling (*S*)- or (*R*)-Fmoc-Asp(OtBu)-OH with *n-*propylamine under standard conditions, followed by *t*Bu-deprotection with trifluoroacetic acid (TFA) (Scheme S6). (*S*)- or (*R*)-2,3-diaminopropionic acid (Dap) was synthesized using a Hofmann rearrangement of *N-*protected (*S*)- or (*R*)-Asn in solution or in the solid phase (Scheme S4) by means of PhI(OCOCF_3_)_2_ (PIFA) and pyridine in DMF/H_2_O.

### 4.3. Peptide Synthesis, General Procedures

Sequences **1**, **2**, **5**, **6**, and **9**–**16** were prepared in a solution using EDC**^.^**HCl/HOBt/TEA, in a DCM/DMF ratio of 4:1, at RT for 12 h, or TBTU/HOBt/DIPEA, in a DCM/DMF ratio of 4:1, at RT for 12 h, as the activating agents (Schemes S3–S6). The removal of acid-labile protecting groups was performed with TFA/DCM (1:1) at RT for 1 h. The removal of benzyl ester protecting groups was performed via catalytic hydrogenation with H_2_/Pd/C, in EtOH, at RT for 12 h.

The remaining sequences were prepared in solid phase using polypropylene syringes fitted with a polyethylene porous disc, Wang resin preloaded with Fmoc-Gly, and Fmoc protecting amino acids. For peptide bond formation, the Fmoc–amino acid (2.5 eq.) was activated with TBTU/HOBt/ DIPEA or DCC/HOBt in DCM/DMF at RT for 3 h. Fmoc group deprotection was performed with 20% piperidine/DMF (2x). Peptide cleavage from Wang resin was conducted with a mixture of TFA and scavengers, i.e., TFA/H_2_O/TIS/PhOH (80:10:10 *v*/*v*/*v*), at RT for 2.5 h.

All crude peptides were purified using a semipreparative reverse-phase (RP)-HPLC over a C18 column (General methods) using H_2_O/acetonitrile mixtures containing 0.1% trifluoroacetic acid. Purity was determined to be >95% according to RP HPLC analysis. The structures were confirmed via ESI-MS, ^1^H, ^13^C, and 2D gCosy NMR spectroscopy (General *Methods).* For the spectroscopic characterization of compounds **1**–**8** see [25]; in general, COOH proton signals cannot be detected.

(*R*)-**9**, (*S*)-**10**. ^1^H-NMR (400 MHz, DMSO-*d*_6_) δ 8.98 (s, 1H, ureaNH), 8.30 (dd, *J* = 6.0, 5.6 Hz, 1H, AMPUMP-NH), 8.10 (dd, *J* = 5.6, 5.2 Hz, 1H, β^2^AlaNH), 7.88 (s, 1H, ureaNH), 7.83 (d, *J* = 8.0 Hz, 1H, ArH_6_), 7.39 (d, *J* = 8.0 Hz, 2H, ArH_2′6′_), 7.21–7.09 (m, 4H, ArH_3,5_ + ArH_3′,5′_), 6.93 (dd, *J* = 7.6, 7.2 Hz, 1H, ArH_4_), 4.20 (dd, *J* = 14.8, 6.0 Hz, 2H, PhCH_2_), 3.23–3.06 (m, 4H, β^2^AlaHβ + COCH_2_CO), 2.55–2.50 (m, 1H, β^2^AlaHα + solvent), 2.23 (s, 3H, ArCH_3_), 1.02 (d, *J* = 7.2 Hz, 3H, CH_3_). ^13^C-NMR (101 MHz, DMSO-*d*_6_) δ 173.9, 169.5, 166.0, 152.6, 138.5, 137.4, 132.8, 130.2, 127.7, 127.4, 126.1, 122.6, 120.9, 118.0, 42.5, 42.0, 41.5, 17.9, 15.7. ESI-MS *m*/*z* calcd. for [C_22_H_27_N_4_O_5_]^+^: 427.2, found: 427.2 [M+H]^+^.

(*S*)-**11**, (*R*)-**12**. ^1^H-NMR (400 MHz, DMSO-*d*_6_) δ 8.97 (s, 1H, ureaNH), 8.35 (br t, 1H, AMPUMP-NH), 8.08 (br t, 1H, DapNHβ), 7.94 (d, *J* = 7.2 Hz, 1H, DapNHα), 7.88 (s, 1H, ureaNH), 7.83 (d, *J* = 8.0 Hz, 1H. ArH_6_), 7.39 (d, *J* = 8.0 Hz, 2H, ArH_2′,6′_), 7.14 (m, 4H, ArH_3,5_ + ArH_3′,5′_), 6.93 (dd, *J* = 7.6, 6.8 Hz, 1H, ArH_4_), 4.39–4.32 (m, 1H, DapHα), 4.24–4.17 (m, 2H, PhCH_2_), 3.48–3.23 (m, 2H, DapHβ + solvent), 3.13 (s, 2H, COCH_2_CO), 2.23 (s, 3H, ArCH_3_), 1.86 (s, 3H, Ac). ^13^C-NMR (101 MHz, DMSO-*d*_6_) δ 169.8, 169.5, 152.7, 138.6, 137.5, 132.4, 130.1, 127.7, 127.5, 126.1, 122.6, 121.0, 117.9, 117.9, 52.8, 41.7, 40.5, 22.7, 17.9. ESI-MS *m*/*z* calcd. for [C_23_H_28_N_5_O_6_]^+^: 470.3, found: 470.2 [M+H]^+^.

(*R*)-**13**, (*S*)-**14**. ^1^H-NMR (400 MHz, CDCl_3_/DMSO-*d*_6_, 1:1) δ 8.75 (s, 1H, ureaNH), 7.99–7.91 (m, 2H, β^3^AlaNH + AMPUMP-NH), 7.78 (d, *J* = 8.4 Hz, 1H, ArH_6_), 7.69 (s, 1H, ureaNH), 7.34 (d, *J* = 8.0 Hz, 2H, ArH_2′6′_), 7.13–6.99 (m, 4H, ArH_3,5_ + ArH_3′5′_), 6.86 (dd, *J* = 7.6, 7.2 Hz, 1H, ArH_4_), 4.28–4.13 (m, 3H, β^3^AlaHβ + PhCH_2_), 3.08 (s, 2H, COCH_2_CO), 2.38 (dd, *J* = 14.0, 6.0 Hz, 1H, β^3^AlaHα), 2.25 (dd, *J* = 14.0, 6.4 Hz, 1H, β^3^AlaHα), 2.21 (s, 3H, ArCH_3_), 1.11 (d, *J* = 6.4 Hz, 3H, CH_3_). ^13^C-NMR (101 MHz, CDCl_3_/DMSO-*d*_6_, 1:1) δ 170.9, 153.6, 139.3, 137.9, 133.0, 130.6, 128.5, 128.1, 126.7, 123.3, 121.9, 118.8, 43.6, 42.8, 42.5, 20.7, 18.6. ESI-MS *m*/*z* calcd. for [C_22_H_27_N_4_O_5_]^+^: 427.2, found: 427.2 [M+H]^+^.

(*S*)-**15**, (*R*)-**16**. ^1^H-NMR (400 MHz, DMSO-*d*_6_) δ 9.32 (s, 1H, ureaNH), 8.39 (d, *J* = 7.6 Hz, 1H, AspNH), 8.31 (dd, *J* = 5.2, 5.6 Hz, 1H, AMPUMP-NH), 8.10 (s, 1H, ureaNH), 7.87 (br s, 1H, propylNH), 7.83 (d, *J* = 8.4 Hz, 1H, ArH_6_), 7.40 (d, *J* = 8.4 Hz, 2H, ArH_2′,6′_), 7.18–7.09 (m, 4H, ArH_3,5_ + ArH_3′,5′_), 6.92 (dd, *J* = 7.6, 7.2 Hz, 1H, ArH_4_), 4.57 (dd, *J* = 14.0, 7.6 Hz, 1H, isoAspHα), 4.19 (d, *J* = 5.6 Hz, 2H, PhCH_2_), 3.27–3.10 (m, 2H, COCH_2_CO + solvent), 3.01–2.96 (m, 2H, propylCH_2_), 2.60 (dd, *J* = 15.4, 6.0 Hz, 1H, isoAspHβ), 2.51–2.46 (m, 1H, isoAspHβ), 2.25 (s, 3H, PhCH_3_), 1.43–1.34 (m, 2H, propylCH_2_), 0.83 (t, *J* = 7.6 Hz, 3H, propylCH_3_). ^13^C-NMR (101 MHz, DMSO-*d_6_*) δ 174.5, 170.4, 169.2, 152.8, 138.8, 137.6, 132.2, 130.1, 127.7, 127.4, 126.0, 122.3, 120.8, 117.7, 50.1, 41.7, 40.5, 37.5, 22.2, 18.2, 11.3. ESI-MS *m*/*z* calcd. for [C_25_H_32_N_5_O_6_]^+^: 498.2, found: 498.2 [M+H]^+^.

### 4.4. Cell Adhesion Assays

The assays were performed as previously described [30,46,47]. Briefly, regarding adhesion assays on Jurkat E6.1 or HL-60 cells, black 96-well plates (Corning Costar) were coated overnight at 4 °C with FN or Fg (10 μg/mL) or VCAM-1 (2 μg/mL) in order to investigate α_4_β_1_ integrin-mediated cell adhesion. To avoid any kind of unspecific binding, plates were incubated with 100 μL/well of blocking solution (1% BSA in HBSS) for 30 min at 37 °C. Cells were counted, stained with the CellTracker green CMFDA (12.5 μM, 30 min at 37 °C, Life Technologies Italia, Milan, Italy) and after three washes, were preincubated with increasing concentrations of new compounds (10^−10^–10^−4^ M) or with the vehicle (methanol) for 30 min at 37 °C.

Then, cells were plated (500,000/well) on coated wells and incubated for 30 min at 37 °C. Afterwards nonadherent cells were removed via washing with a blocking solution, and the remaining adhered cells were lysed with 0.5% Triton X-100 in PBS (30 min at 4 °C). Green fluorescence was measured (Ex485 nm/Em535 nm) using an EnSpire Multimode Plate Reader (PerkinElmer, Waltham, MA, USA).

For adhesion assays mediated by α_5_β_1_ integrin, K562 cells were pre-treated with PMA (64.85 nM, Merck Life Science, Milan, Italy) for 48 h to enhance α_5_β_1_ integrin expression. 96-well plates were coated via passive adsorption with FN (10 μg/mL) overnight at 4 °C; then, the following day, plates were blocked by adding 100 μL/well of a blocking solution (1% BSA in PBS) and incubated for 1 h at 37 °C. K562 cells were counted and preincubated with various concentrations (10^−10^–10^−4^ M) of the peptides or with the vehicle for 30 min at RT. Then, K562 cells were plated (50,000 cells/well) and incubated at RT for 1 h. The wells were then washed three times with 1% BSA in PBS, and 50 μL of hexosaminidase substrate was added and incubated at RT for 1 h. After the addition of 100 μL of stopping solution (50 mM glycine and 5 mM EDTA, pH 10.4), plates were read at 405 nm in an EnSpire Multimode Plate Reader.

In both types of adhesion assay, the number of adherent cells was determined via comparison with a standard curve made in the same plate. Experiments were carried out in quadruplicate and repeated at least three times. Data analysis and EC_50_ or IC_50_ values were calculated using GraphPad Prism 9.5.1 (GraphPad Software, San Diego, CA, USA).

### 4.5. LDV-FITC Competitive Binding Assay

The assays were performed as previously described [48] with the following modifications. Jurkat E6.1 cells were resuspended (50,000 cells/sample) in 0.1% BSA in a HEPES buffer (NaCl 110 mM; KCl 10 mM; Glucose 10 mM; MgCl_2_ 1 mM; CaCl_2_ 1.5 mM; HEPES 30 mM; pH 7.4) and pre-incubated with different concentrations (10^−11^–10^−5^ M) of compounds for 20–30 min at RT. Then, LDV-FITC (5 µM) was added to the cells, and they were incubated in the dark for an additional 30 min at RT. At the end of the incubation with LDV-FITC, cells were washed with 0.1% BSA in a HEPES buffer. The FITC fluorescence intensity was measured using an EnSpire Multimode Plate Reader (PerkinElmer, Waltham, MA, USA). The data were plotted as LDV-FITC specific bindings versus the concentration of the competitor, and the data were fitted to a one-site competition equation; the equilibrium dissociation constant K_i_ was calculated from the IC_50_ using the Cheng–Prusoff equation.

### 4.6. HUTS-21 Monoclonal Antibody Binding

Flow cytometry analysis was performed as previously described [20], with the following modifications. Jurkat E6.1 cells were resuspended in 1% BSA in HBSS (50,000 cells/sample); then, FN (10 μg/mL) and two concentrations of the best peptidomimetics (100–1 nM) were added to the cells for 30 min. Thereafter, phycoerythrin (PE)-conjugated, conformational-sensitive HUTS-21 mAb (PE mouse anti-human CD29 antibody, Becton Dickinson Italia, Milan, Italy) (20 μL/sample) was added for 45 min at room temperature; after two washes, Jurkat E6.1 cells were suspended in PBS, and 10,000 cells/sample were acquired in a Guava^®^EasyCyte Flow Cytometer. The data are presented as the mean fluorescence intensity (MFI). Data were normalized with the relative fluorescence for nonspecific binding evaluated by exposing the cells to an isotype control mAb set to 0.

### 4.7. Western Blot Analysis

Western blot analysis was performed as previously described [20,46], with the following modifications. Jurkat E6.1 cells were cultured for 18 h in an RPMI medium containing a reduced amount of FBS (1%); then, 4 × 10^6^ cells were incubated with different concentrations (10^−7^, 10^−8^, 10^−9^ M) of the most effective cyclic peptides at 37 °C for 1 h. For integrin antagonists, JurkatE6.1 cells were subsequently incubated with FN (10 μg/mL) at 37 °C for 30 min. At the end of the incubation period, Jurkat E6.1 cells were lysed on ice using T-PER^®^ (Tissue Protein Extraction Reagent, Life Technologies Italia, Milan, Italy), supplemented with a phosphatase inhibitor cocktail. Protein extracts were quantified using a BCA Protein Assay (Pierce, Rockford, IL, USA). The proteins from the cytoplasmatic extract were denatured at 95 °C for 3 min, then equal amounts of protein samples (30–50 μg) were loaded and separated via 12% SDS-PAGE. Proteins were transferred onto a nitrocellulose membrane (Biorad, Hercules, CA, USA), which was blocked with 5% nonfat milk in a Tris-buffered saline (10 mM Tris-HCl, pH 8, containing 150 mM NaCl) containing 0.1% Tween 20 for 1 h at room temperature. Membranes were stained overnight at 4 °C with anti-actin antibody (1:5000, Merck Life Science, Milan, Italy) or with anti-phospho-ERK1/2, anti-total ERK1/2, anti-phospho-AKT, anti-total-AKT, or anti-phospho-JNK antibodies (1:1000; Cell Signaling Technology, Danvers, MA, USA). The membranes were incubated with anti-rabbit peroxidase-conjugated secondary antibodies at a dilution of 1:8000. Blots were developed with a SuperSignal West Pico chemiluminescent substrate. Protocols for digital image acquisition and analysis have previously been described [49].

### 4.8. Modelling of α_4_β_1_ Integrin Structure

To define the secondary and the tertiary structure of the integrin, homology modelling was performed with the program MODELLER 10.4 [50], using as templates the experimental dimeric structures of integrins α_4_β_7_ and α_5_β_1_ (PDB IDs: 3V4V and 4WK0, respectively). In total, 100 different models were generated and evaluated with the DOPE score. The model with the most favourable score was retained and used in the subsequent step. Details concerning structures used as templates and considered as potential templates are shown in Supporting Information.

To define the correct interface between the α and β subunits, protein–protein docking was performed with the web server HADDOCK 2.4 [51]. Different models were obtained, and the model from the most populated cluster with the favorable HADDOCK score was selected for the next step. Finally, the metal ions containing sites of the β subunit were refined by performing several optimization steps with the program MODELLER 10.4. In each refinement step, 100 models were generated and evaluated with the DOPE score. The model with the best DOPE score was retained and used as the input in the subsequent step. Thereby, it was possible to define the coordination for each metal ion with surrounding residues and water molecules (Supporting Information). The resulting model was used as the α4β1 structure in protein–ligand docking studies.

### 4.9. Protein–Ligand Docking

The initial geometry of each ligand was generated using UCSF Chimera 1.16 [52] and then optimized at the Hartree–Fock 6-31G* level using ORCA 4 [53]. Using a re-docking procedure and analyzing the results with the web server PacDOCK, it was possible to identify UCSF DOCK 6 [54] as the best docking program for this specific system (see Supporting Information). As a result, UCSF DOCK 6 was used to conduct all the docking using the compounds selected as potential inhibitors of α_4_β_1_ integrin.

To prepare these ligands for docking calculation, UCSF Chimera 1.16 was used in combination with the antechamber software (AmberTools23 version) [55]. In particular, with the *DockPrep* tool included in UCSF Chimera, hydrogens and charges (AMBER ff14SB [56] for standard residues and AM1-BCC [57] for non-standard residues) were added. Chimera and this tool were also used to prepare the receptor input file.

The program UCSF DOCK 6 explores a 3D region defined by a cluster of spheres reproducing the negative image of the binding pocket. To define several sets of overlapping spheres, the *sphgen* tool was used, considering all points outside the receptor surface, a maximum sphere radius of 4.0 A, and a minimum of 1.4 Å. To select the spheres that represent the binding site, all the spheres within 10 Å from the MIDAS extending toward the α subunit were selected with the *sphere_selector* tool. The aim is to direct the ligand toward the binding site, rather than all over the receptor.

Initially, the simulations were performed using the grid-based score of UCSF DOCK 6, thus it was necessary to define the receptor grid (a series of grid points with an overall size defined by a box). The program showbox was used to enclose the selected spheres in the grid box, setting the box length value at 5 Å, while the program grid was used to generate the grid on the basis of these spheres.

Different docking experiments with rigid protein and flexible ligands were performed for each ligand, using different conditions to check for convergence in docked poses, in particular docking experiments, where 1000 different orientations were generated for each anchor and pruned on the basis of clustering, keeping 100 different orientations. To match the receptor spheres with the ligand heavy atoms, an automatic matching method was performed. All these docking experiments were processed using the grid-based score as the scoring function. From these first experiments, for each selected compound, 1000 docked poses were generated. Through a cluster analysis using the tool ClusDOCK from the web server PacDOCK with the gromos algorithm, it was possible to identify a reduced number of clusters for each promising ligand. In conclusion, the most representative poses of the most populated and most favorable energy clusters were identified. The best poses of most of the different docking experiments belong to these clusters; a convergence of the docking experiments can therefore be assumed.

To identify the best pose for each ligand that would be able to exhibit a good potency during the in vitro studies and bind to the receptor during the simulations, a re-scoring was performed. In particular, all the most representative docked poses of each ligand were re-scored using the AMBER score implemented in the program UCSF DOCK 6. In addition, to ameliorate the accuracy of these poses, re-scoring simulations were conducted, treating both the ligand and the residues within a distance of 3.5 Å from the ligands as flexible. For each pose, 3000 MD steps and 100 minimization cycles both before and after the MD simulations were conducted.

## Figures and Tables

**Table 1:** Effect of straight hybrid peptides **1**–**8** [25] and the reference compounds BIO1211 and DS-70 [20] on α_4_β_1_ integrin-mediated Jurkat E6.1 cell adhesion to the endogenous ligand FN (10 μg/mL). Data are presented as IC_50_ (nM) ^a^.

Compd	β^2^-Residue	α_4_β_1_/ FN	Compd	β^3^-Residue	α_4_β_1_/ FN
DS-70	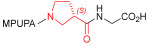	antag ^b^. 4.3 ± 1.7	BIO1211	no	antag. 5.5 ± 4.0
**1**	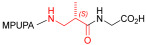	>5000	**5**	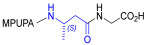	antag. 4060 ± 68
**2**	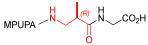	>5000	**6**	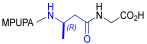	>5000
**3**	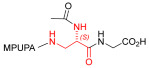	antag. 236 ± 13	**7**	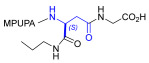	>5000
**4**	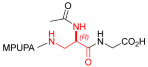	>5000	**8**	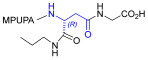	antag. 9.8 ± 1.1

^a^ Values represent the mean ± SD of three independent experiments carried out in quadruplicate. ^b^ Antag. (antagonist) denotes a compound which reduced cell adhesion.

**Table 2:** Effect of partially retro hybrid sequences **9**–**16** and DS-23 [20] on α_4_β_1_ integrin-mediated Jurkat cell adhesion to the endogenous ligand FN (10 μg/mL). Data are presented as IC_50_ (nM) ^a^.

Compd	β^2^-Residue	α_4_β_1_/ FN	Compd	β^3^-Residue	α_4_β_1_/ FN
DS-23	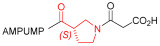	antag ^b^. 51 ± 5		-	
**9**	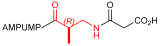	>5000	**13**	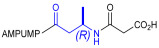	antag ^b^. 416 ± 15
**10**	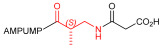	>5000	**14**	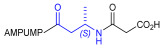	>5000
**11**	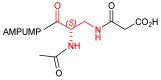	>5000	**15**	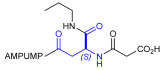	>5000
**12**	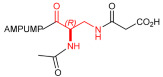	>5000	**16**	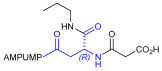	>5000

^a^ Values represent the mean ± SD of three independent experiments carried out in quadruplicate. ^b^ antag. (antagonist) denotes a compound which reduced cell adhesion.

**Table 3:** Selectivity of the hybrid peptides and the reference compound DS-70 [20] toward α_M_β_2_ and α_5_β_1_ integrin, evaluated using cell adhesion assays. Data are presented as IC_50_ (nM) ^a^ for antagonists ^b^ and as EC_50_ for agonists ^c^.

Compd	β-Residue	Jurkat/α_4_β_1_/ VCAM-1	HL60/α_M_β_2_/ Fg	K562/α_5_β_1_/ FN
DS-70	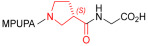	antag ^b^. 5.04 ± 0.51	>5000	>5000
**3**	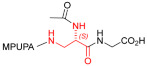	antag. 975 ± 63	antag, 76 ± 13	>5000
**8**	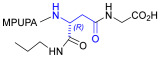	antag. 54 ± 6	>5000	>5000
**13**	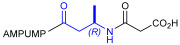	antag. 879 ± 85	Agonist ^c^ 285 ± 36	>5000

^a^ Values represent the mean ± SD of three independent experiments carried out in quadruplicate. ^b^ Antag. (antagonist) denotes a compound which reduced cell adhesion. ^c^ Agonist denotes a compound which increased the number of adherent cells.

**Table 4:** Affinity of the peptides for α_4_β_1_ integrin determined via competitive binding experiments on intact Jurkat E6.1 cells vs. LDV-FITC. Values are expressed as K_i_ (nM) ^a^.^.^

Compd	β-Residue	K_i_
BIO1211	no	6.9 ± 3.1
DS-70	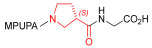	0.94 ± 0.32
**3**	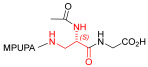	1.3 ± 0.7
**8**	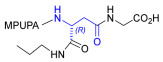	1.9 ± 0.8
**13**	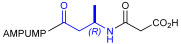	16.7 ± 5.2

^a^ Values represent the mean ± SD of three independent experiments carried out in quadruplicate.

## Data Availability

The data presented in this study are available on request from the corresponding author. The calculated model of α4β1 integrin is freely available at https://site.unibo.it/bioinorgchem/en/downloads.

## Supplementary Materials

The following supporting information can be downloaded at: https://www.mdpi.com/article/10.3390/ijms24119588/s1.
