# Supplementary material for: Design, Pharmacological Characterization, and Molecular Docking of Minimalist Peptidomimetic Antagonists of α4β1 Integrin
Source: Int J Mol Sci. 2023 May 31;24(11):9588. doi: 10.3390/ijms24119588 (PMC10253817; doi:10.3390/ijms24119588)
Supplement: Supplementary file 1 [file ijms-24-09588-s001.zip › ijms-2372512-supplementary.pdf]

Supporting information

Design, Pharmacological Characterization, and Molecular Docking, of Minimalist Peptidomimetic Antagonists of $\alpha_4\beta_1$ Integrin

Monica Baiula[1], Michele Anselmi[2], Francesco Musiani[3], Alessia Ghidini[2], Jacopo Carbone[2], Alberto Caligiana[4], Andrea Maurizio[1], Santi Spampinato[1], Luca Gentilucci[2,5,]*

[1] *Department of Pharmacy and Biotechnology, University of Bologna, via Irnerio 42, 40126 Bologna, Italy.*

[2] *Department of Chemistry "G. Ciamician", University of Bologna, via Selmi 2, 40126 Bologna, Italy.*

[3] *Laboratory of Bioinorganic Chemistry, Department of Pharmacy and Biotechnology, University of Bologna, Viale Fanin 40, 40126 Bologna, Italy.*

[4] *Department of Molecular Biology, Cell Biology and Biochemistry, Brown University, Providence, RI, USA*

[5] *Health Sciences & Technologies (HST) CIRI, University of Bologna, 40064 Ozzano Emilia, Italy*

***** L.G. e-mail: luca.gentilucci@unibo.it; Tel.: +39 0512099570; Fax: +39 0512099456; Web: https://site.unibo.it/mimepept/en; orcid.org/0000-0001-9134-3161.

M.B. Tel.: +39 0512091797; orcid.org/0000-0003-0363-0633.

F.M. Tel.: +39 0512096236; orcid.org/0000-0003-0200-1712.

Contents

Synthetic procedures (Schemes S1-S6) ………………………………………………………… pS1

Cell culture and Concentration-response curves (Figure S1-S4) ………………………… pS6

Molecular Docking Extras (Figures S5-S15 and Tables S1-S3) ………………………… pS8

Synthetic procedures.

The synthesis, isolation, and characterization of compounds **1-8** has been reported elsewhere [1]. For convenience, the synthetic steps are resumed in the synthetic schemes below, together with the synthetic schemes for **9-16**.

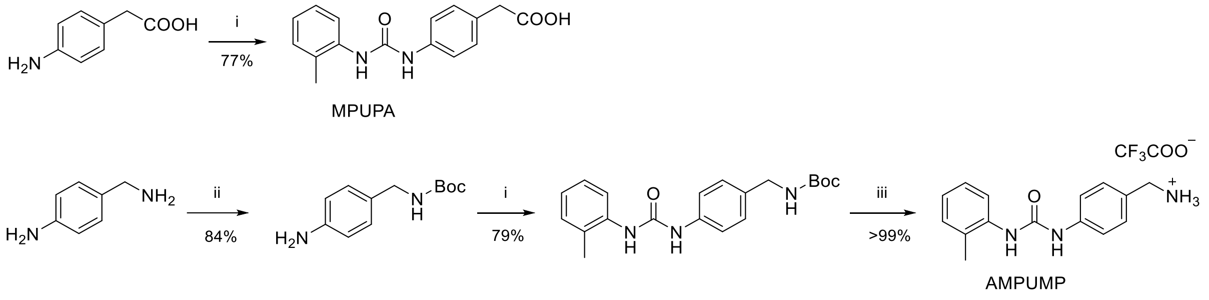

Scheme S1. Synthetic scheme for MPUPA and AMPUMP diphenylureas. *Reagents and conditions:* i) *o*-tolyl isocyanate, DMF, RT, 3 h; ii) Boc_2O, DCM, RT, 2 h; iii) 25% TFA/DCM, RT, 1 h.

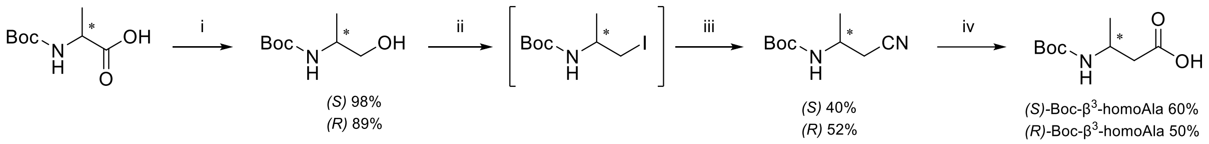

Scheme S2. Synthetic scheme for *N*-Boc β^3-homoAla. *Reagents and conditions:* i) NMM, ethyl chloroformate, THF, 0 °C – RT, 15 min, then $NaBH_{4(aq)}$ 0 °C – RT, 10 min; ii) PPh_3, I_2, imidazole, DCM, reflux, 3 h; iii) KCN, DMSO, 60 °C, 4 h; iv) $KOH_{(aq)}$ 1M/EtOH (1:1), 90 °C, 3 h; or v) HCl 6M, reflux, 12 h.

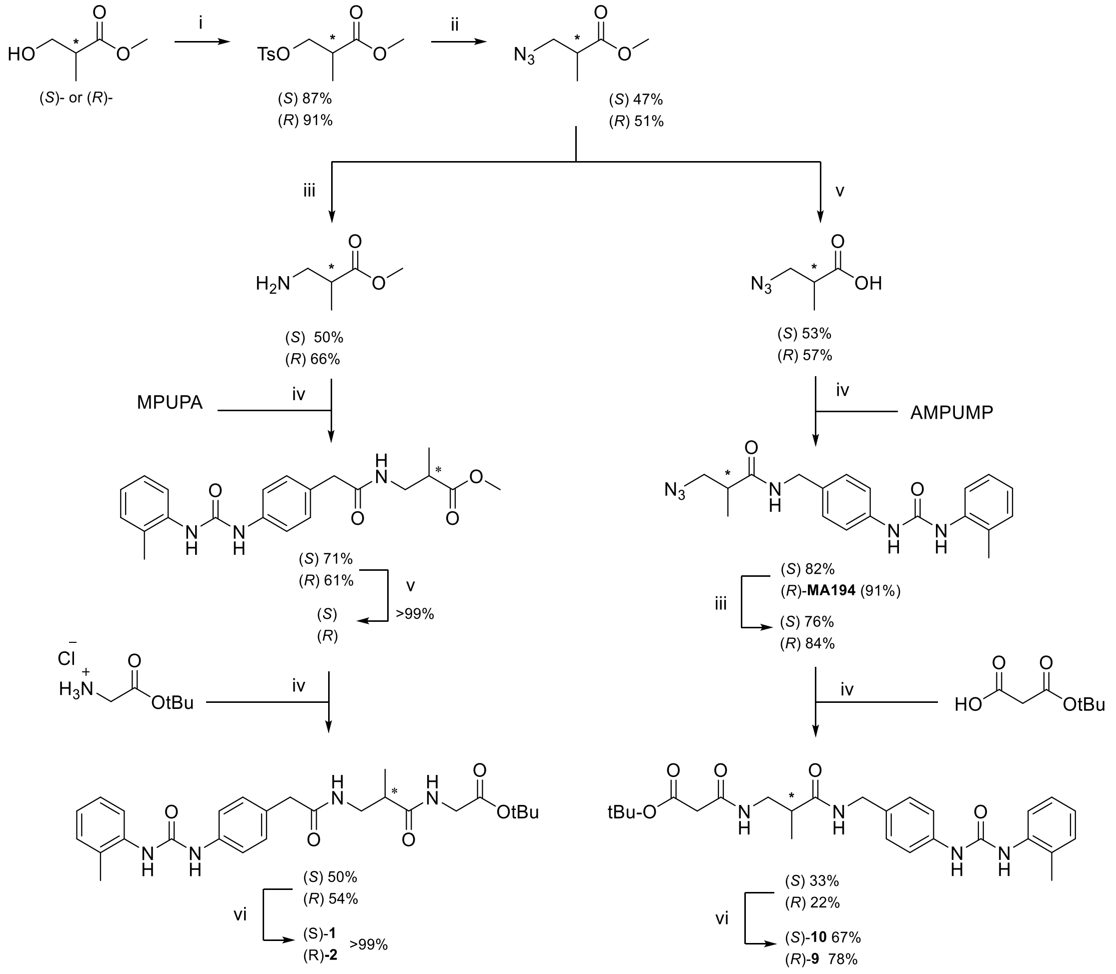

Scheme S3. Synthetic scheme for MPUPA-β^2-homoAla-Gly-OH and malonyl-β^2-homoAla-AMPUMP. *Reagent and conditions:* i) TsCl, TEA, DCM, 0 °C – RT, overnight; ii) NaN_3, DMF, 50 °C, 16 h; iii) H_2, Pd/C, EtOH, RT, 12 h; iv) TBTU, HOBt, DIPEA, DCM/DMF (4:1), RT, 12 h; v) LiOH, THF/H_2O (2:1), 0 °C – RT, 2 h; vi) TFA/DCM (1:1), RT, 1 h.

Method A

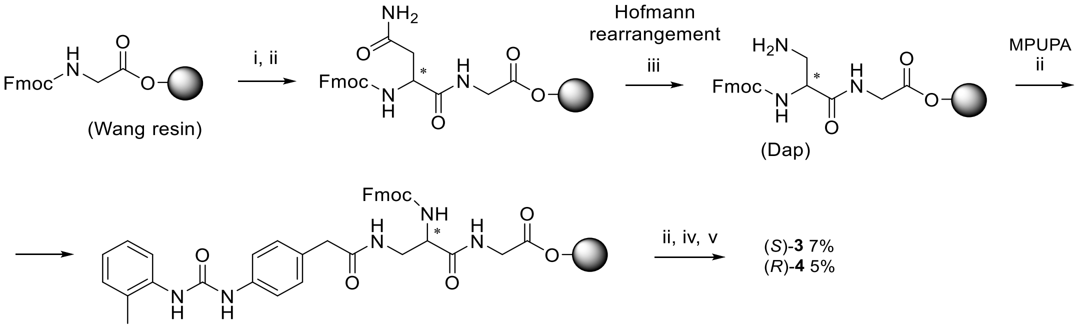

Method B

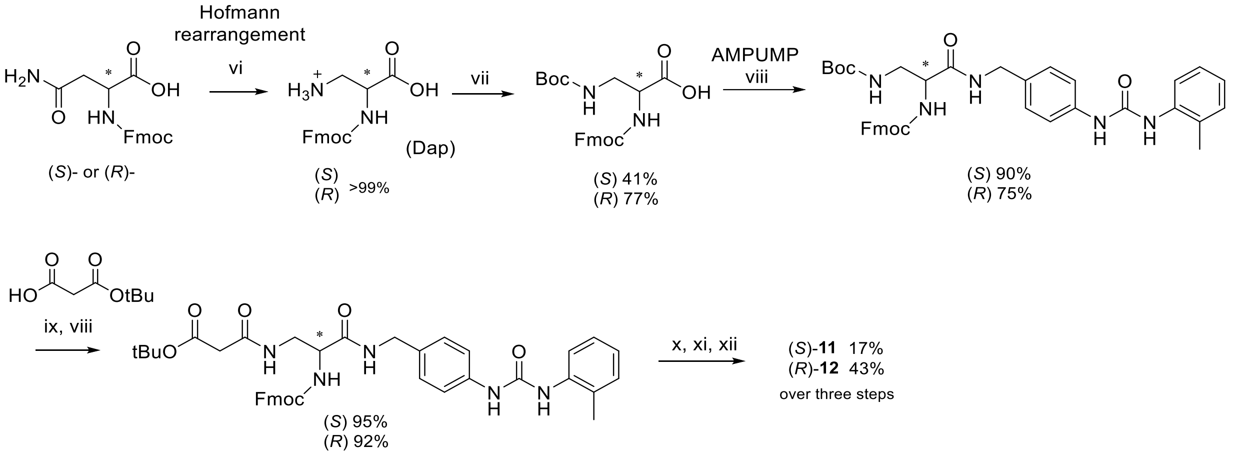

Scheme S4. Synthetic scheme for N_α-Acetyl N_β-MPUPA Dap-Gly-OH and N_α-Acetyl N_β-malonyl Dap-AMPUMP. *Reagents and conditions.* **Method A:** i) 20% piperidine/DMF, RT, 10 min (x2); ii) Fmoc-Asn-OH, DCC/HOBt, DCM/DMF, RT, 3 h; same repeated for MPUPA; iii) PIFA, pyridine, THF/DMF/H_2O (2:2:1), RT, 3 h; iv) Ac_2O, pyridine, DCM, RT, 1 h; v) TFA/H_2O/TIS/PhOH (80:10:10 v/v/v), RT, 2.5 h. *Reagents and conditions.* **Method B:** vi) PIFA, pyridine, DMF/H_2O (2:1), RT, 12 h; vii) Boc_2O, Na_2CO_3, H_2O/dioxane (1:1), RT, 12 h; viii) AMPUMP, TBTU/HOBt/DIPEA, DCM/DMF, RT, 12 h; same repeated for *mono-t*Bu-malonic acid; ix) 25% TFA/DCM, RT, 1 h; x) 20% piperidine/DMF, RT 1 h; xi) acetyl chloride, DIPEA, DCM, 0 °C – RT, 4 h; xii) TFA/DCM (1:1), RT, 1 h.

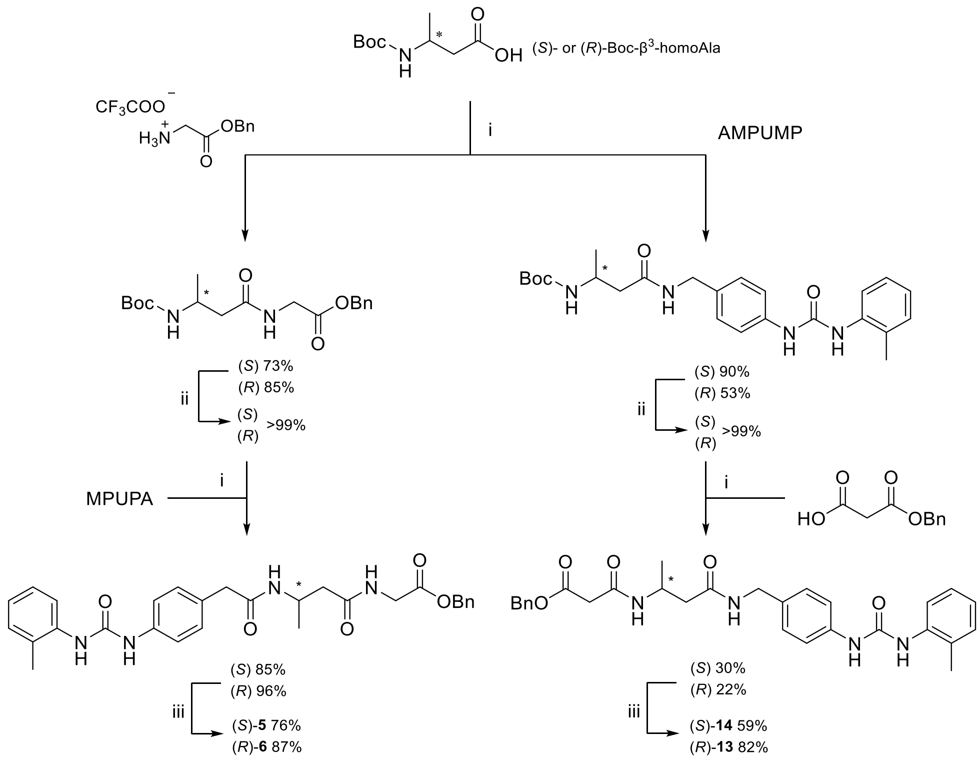

Scheme S5. Synthetic scheme for MPUPA-β^{3}homoAla-Gly-OH and malonyl-β^{3}homoAla-AMPUMP. *Reagent and conditions:* i) EDC·HCl, HOBt, TEA, DCM/DMF (4:1), RT, 12 h; ii) 25% TFA/DCM, RT, 1 h; iii) H_2, Pd/C, EtOH, RT, 12 h.

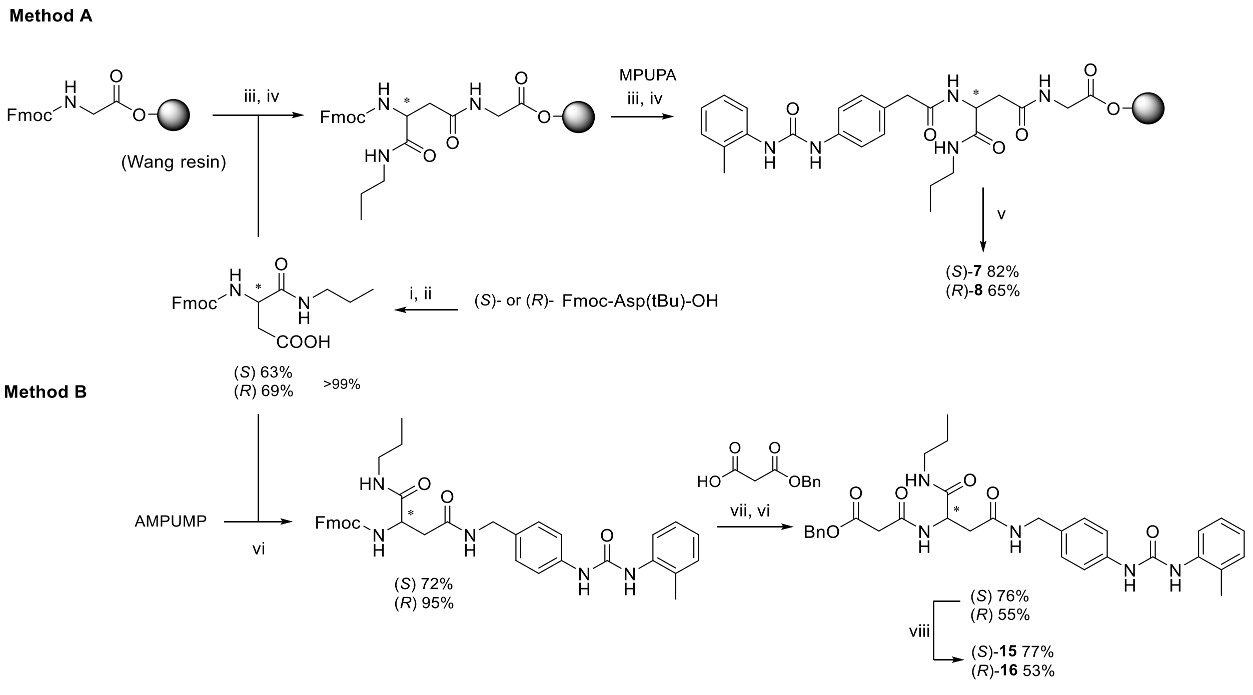

Scheme S6. Synthetic scheme for MPUPA-Asp(Gly)-*n*-propylamine and malonyl-Asp(AMPUMP)-*n*-propylamine. *Reagents and conditions.* **Method A:** i) *n*-propylamine, EDC·HCl/HOBt/TEA, DCM/DMF, RT, 3 h; ii) TFA/DCM (1:1), RT, 1 h; iii) 20% piperidine/DMF, RT, 10 min (x2); iv) Fmoc-Asp-*n*-propylamine, DCC, HOBt, DCM/DMF, RT, 3 h; same repeated for MPUPA; v) TFA/H_2O/TIS/PhOH (80:10:10 v/v/v), RT, 2.5 h. *Reagents and conditions.* **Method B:** vi) EDC·HCl/HOBt/TEA, DCM/DMF, RT, 12 h; same repeated for *mono*-benzylmalonic acid; vii) 20% piperidine/DMF, RT, 1 h; viii) H_2, Pd/C, EtOH, RT, 12 h.

Cell culture. Jurkat E6.1 (expressing both $\alpha_4\beta_1$ and $\alpha_L\beta_2$ integrin), K562 (expressing $\alpha_5\beta_1$ integrin) and HL60 (expressing $\alpha_M\beta_2$ integrin) cell lines were purchased from ATCC (Rockville, MD, USA); these cells were routinely cultured in RPMI-1640 (Life Technologies) supplemented with 1% glutamine and 10% fetal bovine serum (FBS). Cells were grown at 37 °C under 5% CO_2 humidified atmosphere. 40 h before the adhesion assays, K562 and HL60 cells were treated with 25 nM or 40 nM phorbol 12-myristate 13-acetate (PMA; Sigma-Aldrich), respectively, to induced cell differentiation and to increased $\alpha_5\beta_1$ or $\alpha_M\beta_2$ integrin expression on cell surface (Baiula et al., 2016).

Cell adhesion assays. Concentration-response curves, obtained from cell adhesion assays performed in presence of increasing concentrations of peptidomimetics, are shown in the following figures.

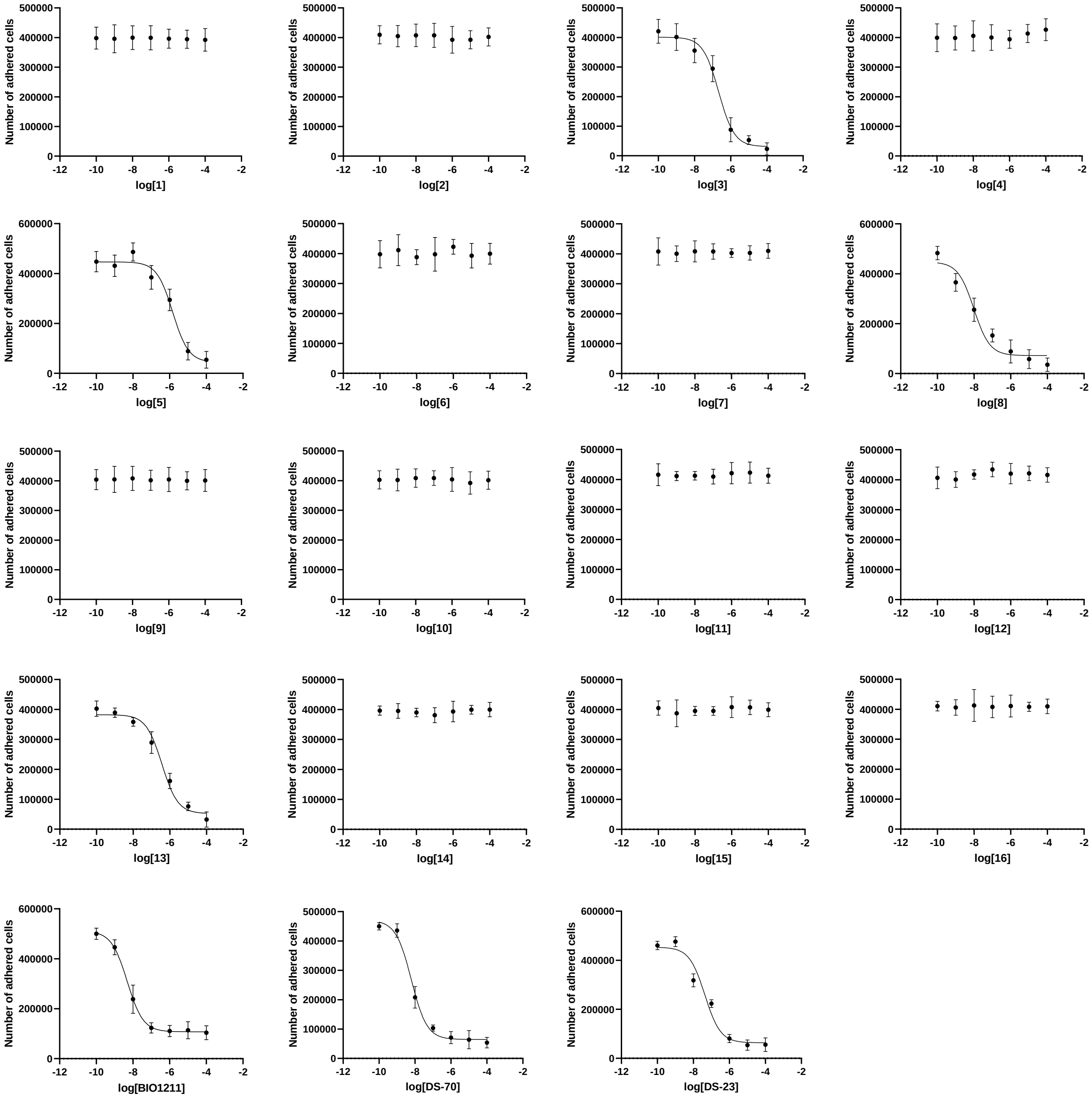

Figure S1. Concentration-response curves obtained from cell adhesion assays: evaluation of peptidomimetic effects on $\alpha_4\beta_1$-mediated Jurkat cell adhesion to FN. Values represent the mean ± SD of at least three independent experiments carried out in quadruplicate.

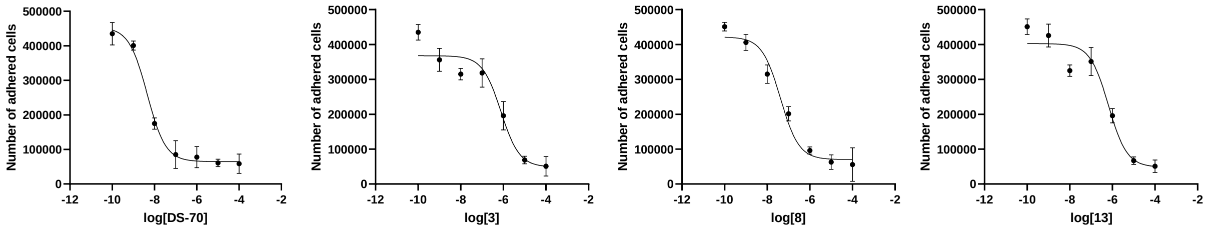

Figure S2. Concentration-response curves obtained from cell adhesion assays: evaluation of peptidomimetic effects on $\alpha_4\beta_1$-mediated Jurkat cell adhesion to VCAM-1. Values represent the mean ± SD of at least three independent experiments carried out in quadruplicate.

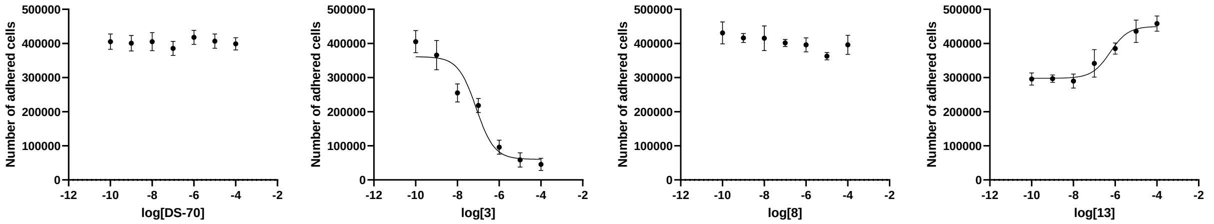

Figure S3. Concentration-response curves obtained from cell adhesion assays: evaluation of peptidomimetic effects on $\alpha_M\beta_2$-mediated HL60 cell adhesion to Fg. Values represent the mean ± SD of three independent experiments carried out in quadruplicate.

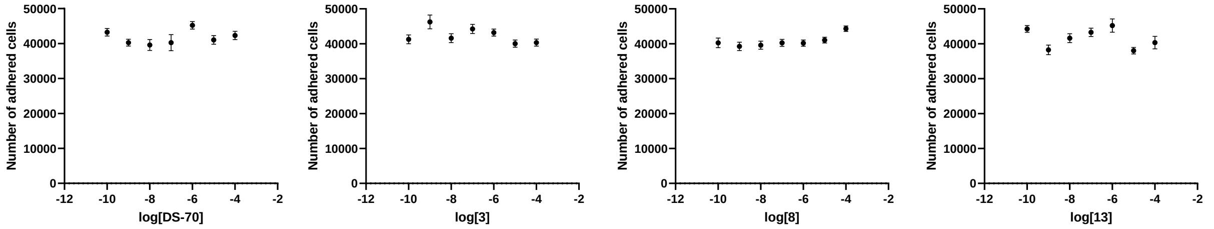

Figure S4 Concentration-response curves obtained from cell adhesion assays: evaluation of peptidomimetic effects on $\alpha_5\beta_1$-mediated K562 cell adhesion to FN. Values represent the mean ± SD of three independent experiments carried out in quadruplicate.

Molecular Docking Extras

Molecular graphics and analyses performed with UCSF Chimera (where not stated otherwise), developed by the Resource for Biocomputing, Visualization, and Informatics at the University of California, San Francisco, with support from NIH P41-GM103311”.

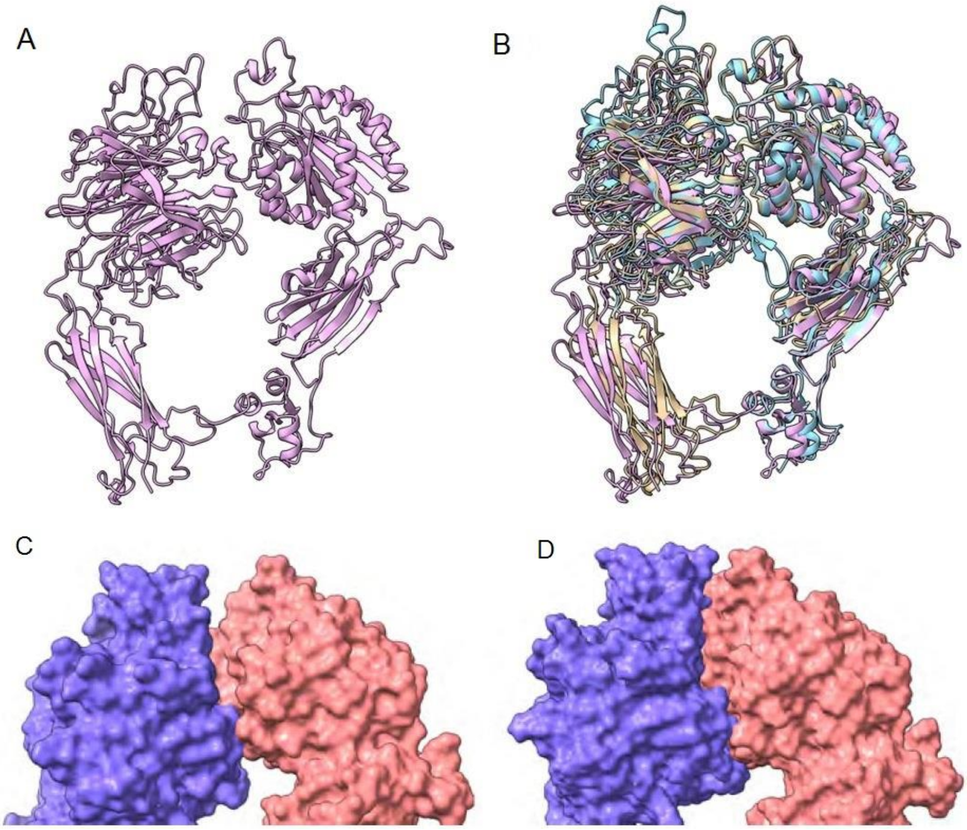

Figure S5. (A) Initial model of $\alpha_4\beta_1$ integrin obtained with MODELLER. (B) Superimposition of the $\alpha_4\beta_1$ model (pink) with the template structures of $\alpha_4\beta_7$ (PDB ID 3V4V, ochre) and $\alpha_5\beta_1$ (PDB ID 4WK0, light blue). (C) Interface of $\alpha_4\beta_1$ before the protein-protein docking. (**D**) Focus on the interface of $\alpha_4\beta_1$ after the protein-protein docking. The surfaces of the α subunit and β subunit are colored in purple and red, respectively.

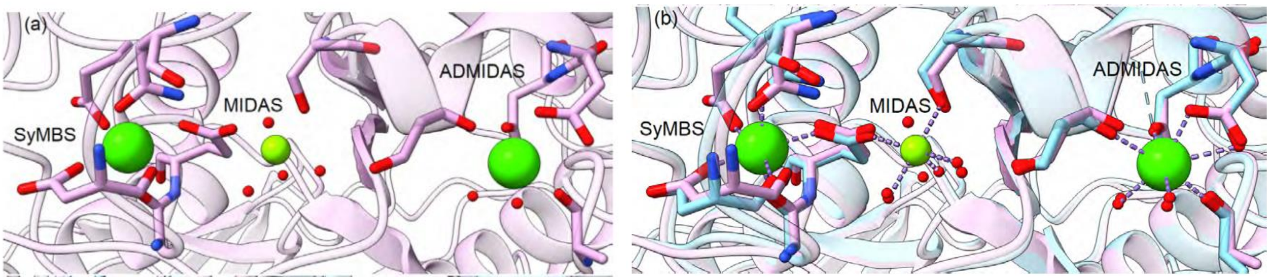

Figure S6. (**a**) MIDAS, ADMIDAS, and SyMBS of the $\alpha_4\beta_1$ model. (**b**) Superimposition between the MIDAS, ADMIDAS, and SyMBS of the β_1 model (pink) and the β_1 experimental structure used as template (PDB ID 4WK0, light blue).

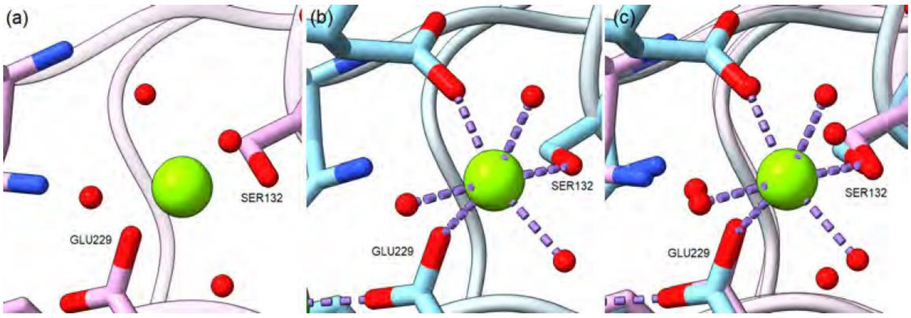

Figure S7. (**a**) Focus on MIDAS of the $\alpha_4\beta_1$ model. (**b**) MIDAS of the experimental structure used as template (4WK0). (**c**) Superimposition between the MIDAS of the model (pink) and of the experimental structure used as template (4WK0, light blue).

Table S1. Metal geometry of the Mg^{2+} ion in the MIDAS of the model (**a**) and the experimental structure used as template (PDB ID 4WK0) (**b**). For each coordinator, the distance to the Mg^{2+} ion is given. In addition, the idealized geometry, coordination number and RMSD value (Å) from that ideal geometry are defined.

(a) Mg^{2+} Coordination		(b) Mg^{2+} Coordination	
**Coordinator**	**Distance (Å)**	**Coordinator**	**Distance (Å)**
GLU229	2.08	GLU229	2.03
SER132	2.16	SER132	2.16
HOH	2.03	Ligand	2.04
HOH	2.06	HOH	2.03
HOH	2.06	HOH	2.08
HOH	2.08	HOH	2.11
**Geometry**	octahedron	**Geometry**	octahedron
**Coordination**	6	**Coordination**	6
**RMSD**	0.007	**RMSD**	0.212

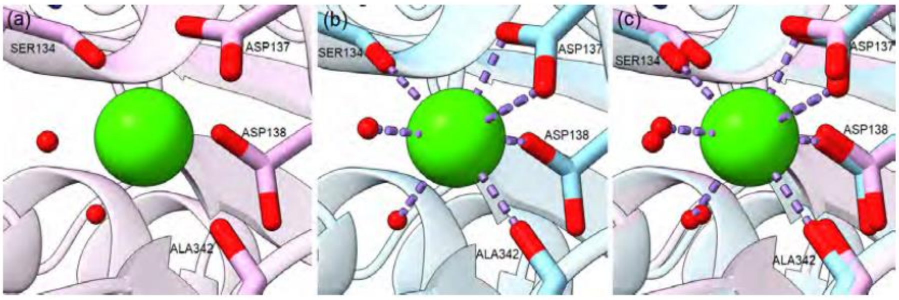

Figure S8. (**a**) Focus on ADMIDAS of the $\alpha_4\beta_1$ model. (**b**) ADMIDAS of the experimental structure used as template (4WK0). (**c**) Superimposition between the ADMIDAS of the model (pink) and of the experimental structure used as template (4WK0, light blue).

Table S2. Metal geometry of the Ca^{2+} ion in the ADMIDAS of the model (**a**) and the experimental structure used as template (PDB ID 4WK0) (**b**). For each coordinator, the distance to the Mg^{2+} ion is given. In addition, the idealized geometry, coordination number and RMSD value (Å) from that ideal geometry are defined.

(a) Ca^{2+} Coordination		(b) Ca^{2+} Coordination	
**Coordinator**	**Distance (Å)**	**Coordinator**	**Distance (Å)**
ASP137	2.41	ASP137	2.36
ASP137	3.02	ASP137	2.86
ASP138	2.41	ASP138	2.37
SER134	2.38	SER134	2.53
ALA342	2.59	ALA342	2.47
HOH	2.30	HOH	2.32
HOH	2.46	HOH	2.46
**Geometry**	pentagonal bipyramid	**Geometry**	pentagonal bipyramid
**Coordination**	7	**Coordination**	7
**RMSD**	0.492	**RMSD**	0.422

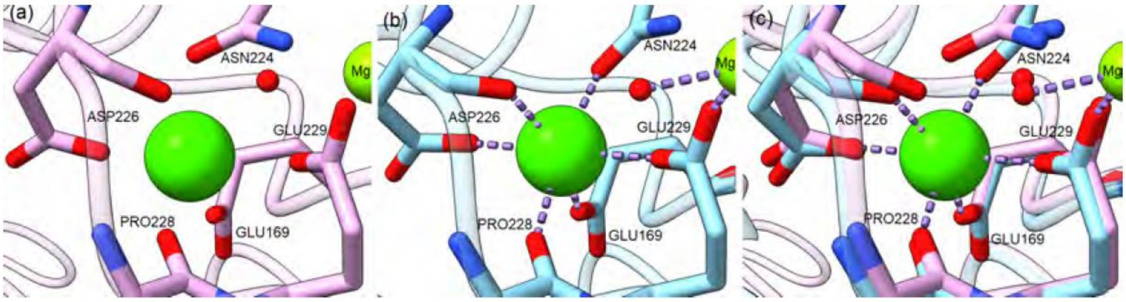

Figure S9 (**a**) Focus on SyMBS of the $\alpha_4\beta_1$ model. (**b**) SyMBS of the experimental structure used as template (4WK0). (**c**) Superimposition between the SyMBS of the model (pink) and of the experimental structure used as template (4WK0, light blue).

Table S3. Metal geometry of the Ca^{2+} ion in the SyMBS of the model (**a**) and the experimental structure used as template (PDB ID 4WK0) (**b**). For each coordinator, the distance to the Mg^{2+} ion is given. In addition, the idealized geometry, coordination number and RMSD value (Å) from that ideal geometry are defined.

(a) Ca^{2+} Coordination		(b) Ca^{2+} Coordination	
**Coordinator**	**Distance (Å)**	**Coordinator**	**Distance (Å)**
ASN224	2.43	ASN224	2.29
GLU169	2.36	GLU169	2.40
GLU229	2.50	GLU229	2.34
PRO228	2.16	PRO228	2.26
ASP226 O	2.27	ASP226 O	2.34
ASP226 OD1	2.15	ASP226 OD1	2.22
**Geometry**	octahedron	**Geometry**	octahedron
**Coordination**	6	**Coordination**	6
**RMSD**	0.244	**RMSD**	0.243

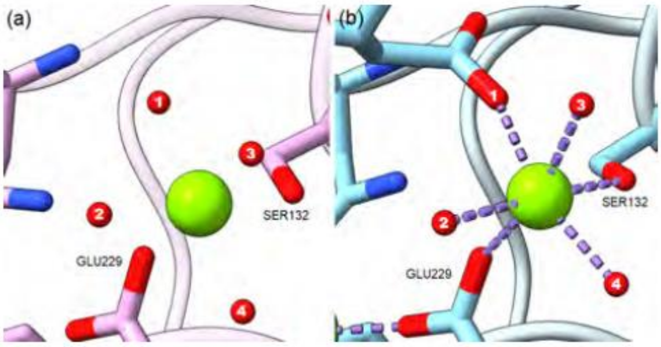

Figure S10. (**a**) Potential positions of ligands in the primary coordination sphere of the metal in the model (pink) compared to (**b**) the position occupied by the experimental ligand in the structure used as template (4WK0, light blue).

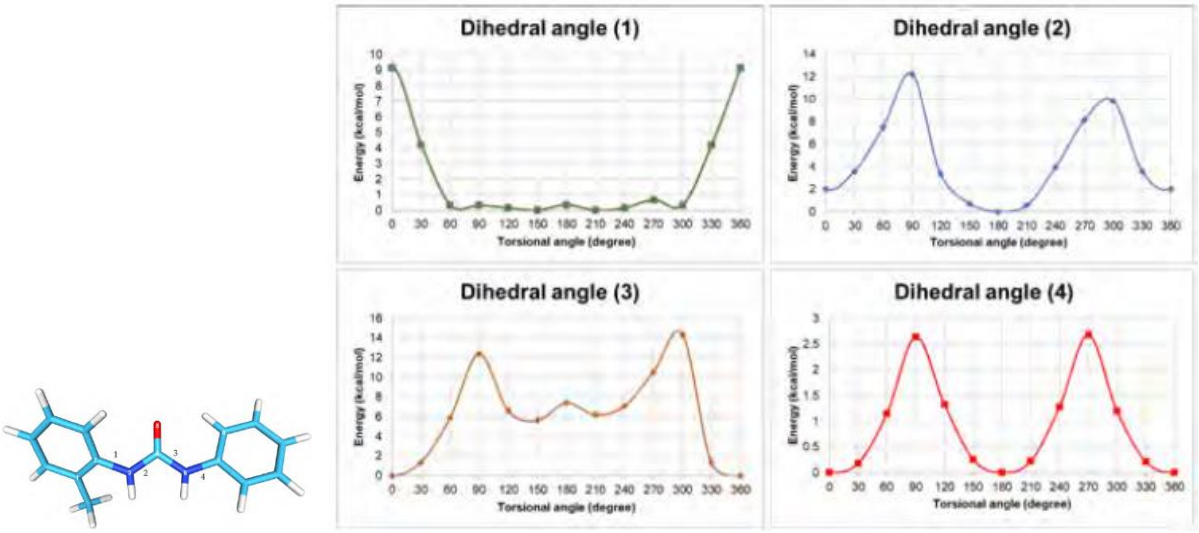

Figure S11. Optimized structure of N-(2-methylphenyl)-N-phenylurea used in relaxed surface scans. Carbon atoms are colored in light blue, while other atoms are colored according to atom type. Carbon atoms are colored in light blue, while other atoms are coloured according to atom type. Rotational potential energy profiles for N-(2-methylphenyl)-N-phenylurea around the 4 different dihedral angles scanned.

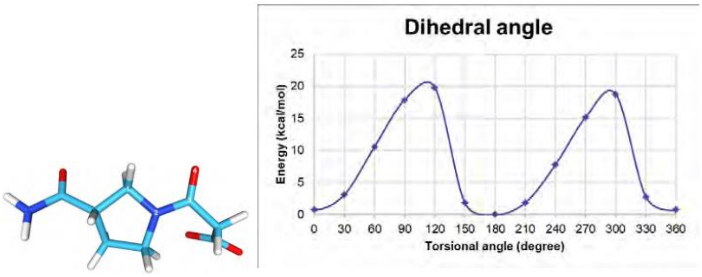

Figure S12. Optimized structure of β-Pro moiety used in relaxed surface scan. Carbon atoms are colored in light blue, while other atoms are colored according to atom type. Rotational potential energy profiles for β-Pro moiety around the amidic bond.

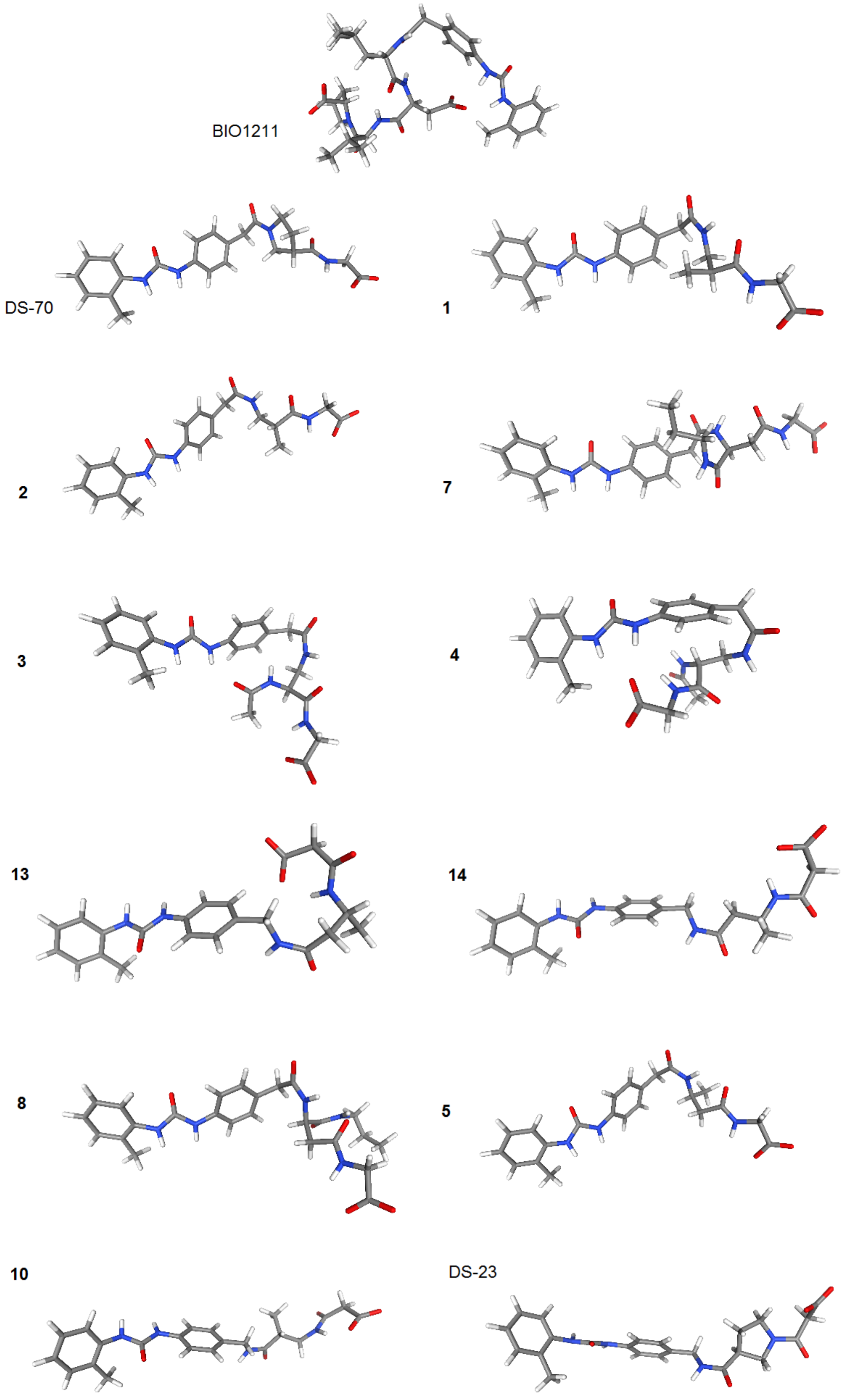

Figure S13. HF 6-31G* optimized structure of compounds used in protein-ligand docking experiments.

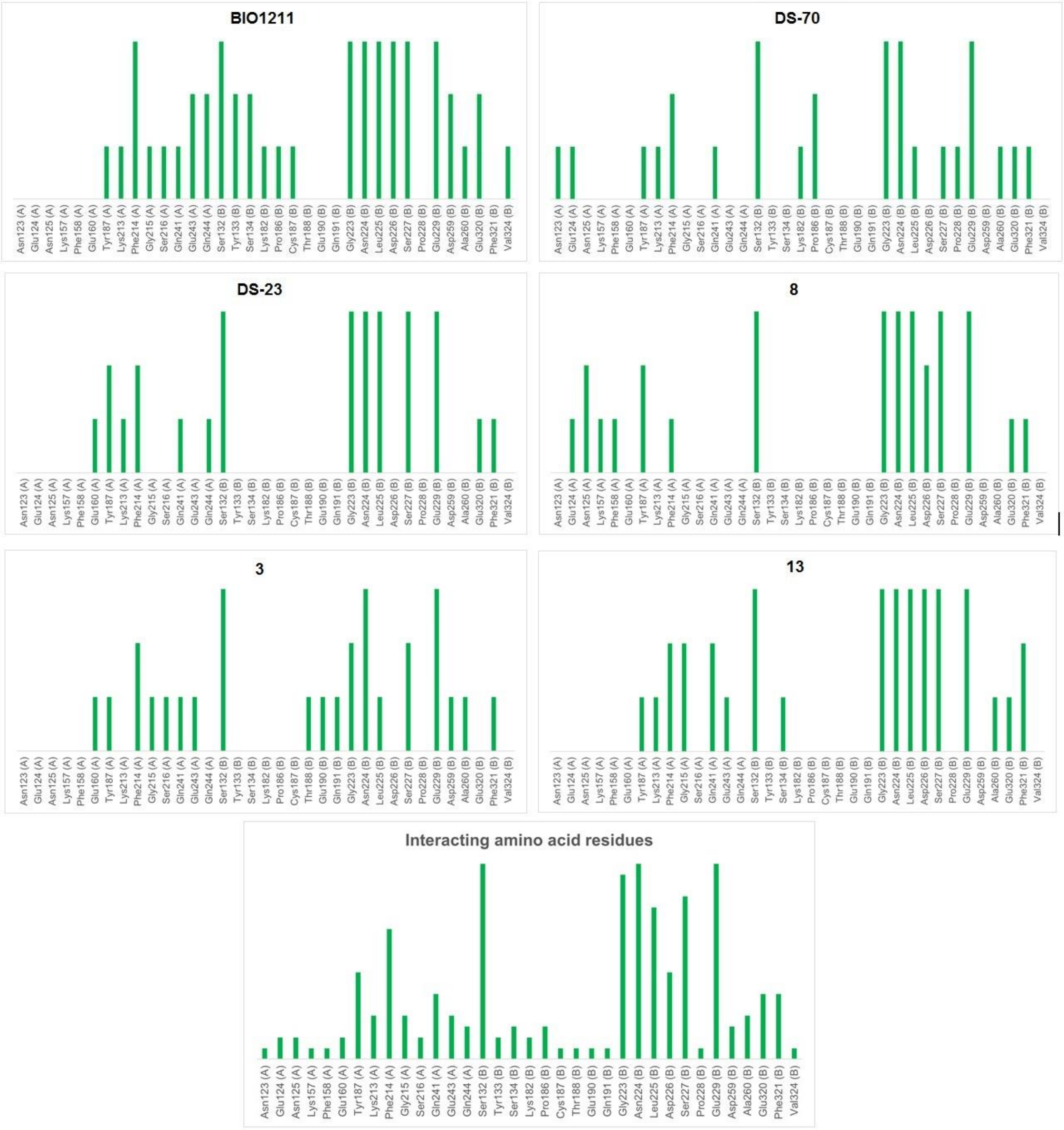

Figure S14. $\alpha_4\beta_1$ integrin residues involved in interaction with the best docked poses of the ligands BIO1211, DS-23, DS-70, **8**, **3**, **13**.

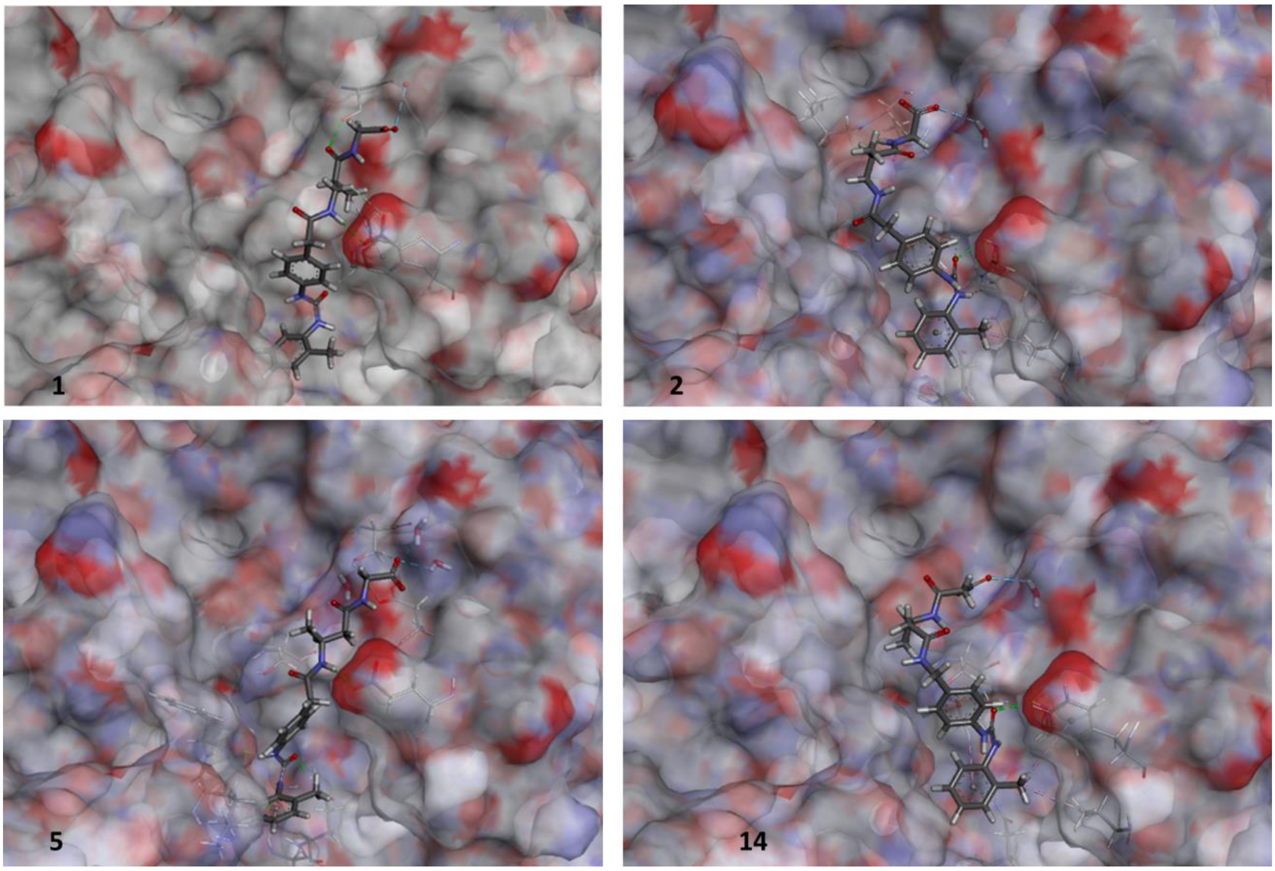

Figure S15. Calculated binding conformations of hybrid ligands within the $\alpha_4\beta_1$ integrin binding site. Structures with poses similar to those of the ligands shown in Figure 5 have been selected for comparison. In general, distances between carboxylate of the ligands and MIDAS are > 3 Å. Ligands are rendered in stick and colored by atoms. The integrin binding site is represented by its partially transparent, solid solvent-accessible surface, colored by the atomic interpolated charge. Key receptor residues are represented in tiny sticks, and nonbonding interactions are indicated as dashed lines. Images were obtained using BIOVIA DSV2021.

1. Anselmi, M.; Baiula, M.; Santino, F.; Zhao, J.; Spampinato, S.; Calonghi, N.; Gentilucci, L. Design of α/β-hybrid peptide ligands of α4β1 integrin equipped with a linkable side chain for chemoselective biofunctionalization of microstructured materials, *Biomedicines*, 2021, **9**, 1737.
